# MAOB promotes ROS-mediated DNA damage, triggering a cyclic MAOB-HNF1A-53BP1-p53 axis that suppresses the malignancy of clear cell renal cell carcinoma

**DOI:** 10.1016/j.redox.2025.103945

**Published:** 2025-11-25

**Authors:** Kuo-Hao Ho, Yung-Wei Lin, Hsiang-Ching Huang, Feng-Ru Lai, Yi-Chieh Yang, Chung-Howe Lai, Yu-Ching Wen, Feng-Koo Hsieh, Wei-Jiunn Lee, Ming-Hsien Chien

**Affiliations:** a Department of Biochemistry and Molecular Cell Biology, School of Medicine, College of Medicine, Taipei Medical University, Taipei, Taiwan; b Graduate Institute of Clinical Medicine, College of Medicine, Taipei Medical University, Taipei, Taiwan; c Department of Urology, Wan Fang Hospital, Taipei Medical University, Taipei, Taiwan; d Department of Urology, School of Medicine, College of Medicine and TMU Research Center of Urology and Kidney (TMU-RCUK), Taipei Medical University, Taipei, Taiwan; e International Master/PhD Program in Medicine, College of Medicine, Taipei Medical University, Taipei, Taiwan; f School of Oral Hygiene, College of Oral Medicine, Taipei Medical University, Taipei, Taiwan; g The Genome Engineering & Stem Cell Center, School of Medicine, Washington University, St. Louis, USA; h Department of Medical Education and Research, Wan Fang Hospital, Taipei Medical University, Taipei, Taiwan; i TMU Research Center for Cancer Translational Medicine, Taipei Medical University, Taipei, Taiwan; j Pulmonary Research Center, Wan Fang Hospital, Taipei Medical University, Taipei, Taiwan; k Traditional Herbal Medicine Research Center, Taipei Medical University Hospital, Taipei, Taiwan

**Keywords:** MAOB, ROS, p53, HNF1A, 53BP1, ccRCC

## Abstract

Monoamine oxidases (MAOA and MAOB) are mitochondrial enzymes that degrade various monoamine neurotransmitters, which have been recognized as important regulators of tumor progression. Recently, conflicting roles of both enzymes were identified in several cancer types. However, their potential involvement in the progression of clear cell renal cell carcinoma (ccRCC) remains unclear. In this study, *in silico* analysis of the TCGA-KIRC dataset revealed that MAOB has a more significant prognostic impact than MAOA and serves as an independent prognostic factor for overall survival in ccRCC. Lower MAOB transcript and protein levels were observed in RCC tissues compared to normal tissues and were associated with larger tumor sizes. Enzymatically active MAOB promoted reactive oxygen species (ROS)-induced DNA damage, subsequently enhancing the stability and transcriptional activity of p53, which induced G1 cell cycle arrest, mitochondria apoptosis, and lipid peroxidation-triggered ferroptosis, ultimately suppressing tumor growth both in vitro and in vivo. Molecular studies showed that MAOB stabilizes and activates p53 through post-translational modifications (PTMs), including increased phosphorylation at Ser15 and acetylation at Lys382, as well as activation of the hepatocyte nuclear factor 1 homeobox A (HNF1A)–p53-binding protein 1 (53BP1) axis. Activated p53, in turn, regulated MAOB through positive feedback. Clinically, ccRCC samples revealed a positive correlation between MAOB and HNF1A expression, with patients expressing high levels of both having the best prognoses. Regarding therapeutic aspects, we discovered that DNA methyltransferase inhibitors serve as potential MAOB inducer in ccRCC. The current findings reveal novel mechanisms by which MAOB suppresses the malignancy of ccRCC and suggest that MAOB may serve as a valuable prognostic marker in the management of ccRCC.

## 1 Introduction

Renal cell carcinoma (RCC) is the deadliest among the urologic cancers and can be categorized into clear cell RCC (ccRCC), which represents 70 %–80 % of cases, papillary RCC (pRCC) at around 15 %, and chromophobe RCC (chRCC) at approximately 5 % [1,2]. Due to its asymptomatic nature, 25 % of patients are initially diagnosed with advanced metastatic disease (mRCC), and around 20 %–40 % of those with localized RCC eventually develop metachronous metastases [3]. Therapeutic strategies for mRCC have undergone a significant transformation in recent years, evolving from cytokine treatments to vascular endothelial growth factor (VEGF)-VEGF receptor (VEGFR)-targeted therapies and immune checkpoint blockade. Despite these advancements, advanced RCC continues to be a deadly disease [4]. Somatic mutations or methylation of the von Hippel-Lindau (*VHL*) tumor-suppressor gene are among the most well-defined and frequently observed alterations in ccRCC. *VHL* gene alterations are recognized as prognostic biomarkers in stage I–III ccRCC patients following a nephrectomy [5]. However, in other studies, *VHL* gene alterations were reported to hold no prognostic or predictive significance in patients with ccRCC [6]. To date, clinically useful biomarkers for predicting mRCC progression remain scarce, aside from VHL [7]. Therefore, it is crucial to uncover new molecular mechanisms driving RCC development and identify reliable markers for predicting disease progression and clinical outcomes in RCC patients.

Recent evidence indicates that cancer cells exploit neurotransmitter-initiated signaling pathways to activate uncontrolled proliferation and dissemination [8]. The monoamine oxidase (MAO) enzyme family, consisting of MAOA and MAOB, is located on the outer mitochondrial membrane and was initially known for catalyzing the oxidative deamination of monoamine neurotransmitters in the system, playing a role in various neurological disorders [9]. Recent studies revealed that MAOs play conflicting roles in tumor progression across different cancers. For instance, elevated levels of MAOA were observed in non-small cell lung cancer (NSCLC) compared to non-tumor tissues, with MAOA expression showing positive correlations with clinical stages and lymph node metastasis [10]. In prostate cancer (PCa) and NSCLC, MAOA was shown to promote the epithelial-to-mesenchymal transition (EMT) and metastasis by stabilizing hypoxia-inducible factor 1α (HIF1α) [11,12]. Additionally, Wang et al. demonstrated that MAOA drives immunosuppressive polarization of tumor-associated macrophages (TAMs) by increasing oxidative stress [13]. In contrast to its oncogenic roles, in hepatocellular carcinoma (HCC), MAOA was linked to better patient prognoses and was found to suppress metastasis by inhibiting the adrenergic system and its transactivation of epidermal growth factor receptor (EGFR) signaling [14]. Furthermore, downregulation of MAOA due to hypermethylation was noted in cholangiocarcinomas, where MAOA overexpression was shown to inhibit tumor growth and invasion [15]. Compared to MAOA, fewer studies have addressed the role of MAOB in cancer except for colorectal carcinoma (CRC) [16], gliomas [17], PCa [18], and head and neck cancers [19,20], in which cases diverse roles were displayed.

In this study, we analyzed the prognostic significance of both MAOA and MAOB in ccRCC using The Cancer Genome Atlas Kidney Renal Clear Cell Carcinoma (TCGA-KIRC) cohort. Our findings revealed that genes associated with MAOB showed considerably greater clinical relevance compared to those associated with MAOA in ccRCC. A significant downregulation of MAOB levels was observed in ccRCC tissues, and this was correlated with advanced clinical stages, larger tumor sizes, and poor prognoses. Further study indicated that MAOB enzymatically increased reactive oxygen species (ROS)-induced DNA damage, leading to increased stability and transcriptional activity of p53. This, in turn, triggered G_1_ cell-cycle arrest, mitochondrial apoptosis, and lipid peroxidation-driven ferroptosis, ultimately inhibiting tumor growth in vitro and in vivo. Molecular studies revealed that MAOB stabilizes and activates p53 via post-translational modifications (PTMs), including increased Ser15 phosphorylation and Lys382 acetylation, and reduced ubiquitination. Additionally, MAOB enhances p53 activation and expression through the hepatocyte nuclear factor 1A (HNF1A)-p53-binding protein 1 (53BP1) axis. Thus, MAOB has become a promising biomarker for predicting ccRCC progression, and enhancing its expression or enzymatic activity could offer a novel therapeutic strategy for ccRCC.

## 2 Methods

### 2.1 Data collection from bioinformatics analyses

To conduct a comprehensive analysis of MAOA and MAOB expressions and their clinical importance in RCC patients, we employed several methodologies and datasets. TCGA RNA-sequencing (RNA-Seq) data for ccRCC and pRCC patients were obtained from UCSC Xena (https://xena.ucsc.edu/). A Spearman correlation analysis was conducted to identify genes associated with MAOA and MAOB expressions in ccRCC. Genes with a correlation coefficient of >0.3 were classified as positively correlated, while those with a correlation coefficient of < -0.3 were classified as negatively correlated. Subsequently, a Cox regression survival analysis was conducted on MAOA/MAOB-associated genes using data from TCGA ccRCC cohort. Gene candidates with a hazard ratio (HR) of >1 and a *p* value of <0.05 were identified as markers of a poor prognosis, whereas those with an HR of <1 and a *p* value of <0.05 were considered markers of a good prognosis. Genes with a *p* value of ≥0.05 were deemed non-prognostic. For the survival analysis, we included patients with complete information on pathological stage, tumor size (T), lymph node involvement (N), and metastasis (M). Patients were stratified into high- and low-expression groups based on the median gene expression level. A log-rank test was performed to assess the statistical significance of differences in overall survival (OS) and disease-specific survival (DSS) between these groups. Additionally, a multivariate Cox regression analysis was performed to evaluate whether MAOB or HNF1A served as an independent prognostic predictor, considering patients' T/N/M and pathological stages. To analyze the correlation of MAOB or HNF1A expression with different tumor sizes, a Wilcoxon signed-rank test was performed to determine statistical differences. Additionally, a Chi-squared test was conducted to assess the association between high- and low-expression MAOB/HNF1A groups and various clinical variables, including gender, pathological stages, and T/N/M stages. To compare protein levels of MAOB and HNF1A between tumor and normal tissues, proteomic datasets of ccRCC patients were obtained from Clinical Proteomic Technology Assessment for Cancer (CPTAC), and statistical differences were assessed using the Wilcoxon signed-rank test.

### 2.2 Immunohistochemical (IHC) staining

An established human RCC tissue microarray (TMA) (BC07115a) was obtained from US Biomax (Rockville, MD, USA). Paraffin-embedded RCC tissue or tumor nodule sections (4 μm thick) on coated slides were first washed with xylene to remove the paraffin, then rehydrated through a series of alcohol dilutions and rinsed with phosphate-buffered saline (PBS; pH 7.2). After a 24-h incubation at 4 °C with primary antibodies against MAOB, p53, or proliferative genes, slides were thoroughly washed three times with PBST. Signal development was carried out using the conventional streptavidin peroxidase method (LSAB Kit K675; DAKO, Copenhagen, Denmark). The H-score method was applied to quantify the staining intensity of the IHC results. Scores ranged from 0 (no staining) to 3 (strong staining).

### 2.3 Cell culture

The human 786-O, A498, and Caki-1 ccRCC cell lines, as well as kidney tubular epithelial HK2 cells, were sourced from American Type Culture Collection (Manassas, VA, USA). Caki-1 and A498 cells were cultured in minimum essential medium (MEM) (Gibco, Grand Island, NY, USA), 786-O cells in Royal Park Memorial Institute (RPMI) 1640 (Gibco), and HK2 cells in Dulbecco's modified Eagle medium (DMEM)/F12, supplemented with 10 % fetal bovine serum, 100 units/mL penicillin, 100 μg/mL streptomycin, and 1 % glutamine, all maintained at 37 °C in a 5 % CO_2_ humidified environment.

### 2.4 Antibodies and reagents

Selegiline (M003), pargyline (P8013), rasagiline (SML0124), N-acetyl-l-cysteine (NAC; A7250), glutathione (G6013), cycloheximide (01810), 5-aza-2′-deoxycytidine (5-Aza; 189825), 2′,7′-dichlorofluorescin diacetate (DCFDA; 287810), and JC-1 (420200) were purchased from Sigma-Aldrich (St. Louis, MO, USA). The antibodies are detailed in Table S1.

### 2.5 Western blot analysis

Cells were harvested and lysed with PRO-PREPTM protein extraction buffer (iNtRON, Gyeonggi-do, Korea) containing a protease inhibitor cocktail. Protein extracts (40 μg) were separated by sodium dodecylsulfate polyacrylamide gel electrophoresis (SDS-PAGE), transferred to a polyvinylidene difluoride (PVDF) membrane (Bio-Rad, Hercules, CA, USA), and blocked with 5 % bovine serum albumin in 0.1 % TBST. The membranes were incubated overnight at 4 °C with the indicated primary antibodies, followed by horseradish peroxidase (HRP)-conjugated secondary antibodies. Protein expression signals were visualized using enhanced chemiluminescence (ECL) reagents (Pierce Biotechnology, Rockford, IL, USA) and detected with the MultiGel-21 chemiluminescence imaging system (TOP BIO, New Taipei City, Taiwan).

### 2.6 Construction of the Y435W mutation of MAOB

The pLX304-MAOB plasmid was sourced from the DNASU Plasmid Repository (https://dnasu.org/DNASU/Home.do) and utilized as a template. A MAOB-Y435W mutant construct was created using the QuickChange® Lightning Site-Directed Mutagenesis Kit (Agilent Technology, La Jolla, CA, USA). To introduce the mutation, a specific primer pair (5′-CACTGGAGCGGCTGGATGGAGGGGGCTG-3′ and 5′-CAGCCCCCTCCATCCAGCCGCTCCAGTG-3′) was used. Mutant DNA was amplified with Pfu Ultra II DNA polymerase, and *Dpn*I endonuclease was applied to digest the non-mutated plasmids. The plasmid carrying the mutated *MAOB* gene was then transformed into *Escherichia coli* DH5α for amplification and isolation. Sequencing was performed to confirm the successful mutation.

### 2.7 Establishment of gene knockdown (KD) and overexpression of RCC cells

To knock down the indicated genes (*MAOB*, *p53*, *53BP1*, and *HNF1A*) or overexpress MAOB, lentiviral particles containing the respective short hairpin (sh)RNAs or pLX304-MAOB were generated and used to infect RCC cells for 24 h, following protocols from our previous study [21]. shRNAs targeting specific genes were obtained from the National RNAi Core Facility at Academia Sinica (Taipei, Taiwan), with the targeting sequences listed in Table S2.

### 2.8 Cell-proliferation and colony-forming assays

MAOB-manipulated RCC cells were seeded in 96-well plates (3 × 10^3^ cells/well) or 10-cm dishes (2 × 10^5^ cells/dish) containing complete medium for the indicated times, and then subjected to a CCK-8 cell proliferation assay (Sigma-Aldrich, St. Louis, MO, USA) and a trypan blue dye exclusion assay, following protocols from our previous study [22]. For long-term effects, MAOB-manipulated RCC cells were plated in six-well plates at a density of 10^3^ cells/well and cultured for 7–10 days. Cells were then fixed with methanol and stained with 1 % crystal violet, and colony numbers were manually counted using ImageJ software (National Institutes of Health, Bethesda, MD, USA).

### 2.9 Spheroid-forming assay

RCC cells manipulated for MAOB (5 × 10^3^ cells/well) were seeded into ultra-low attachment six-well plates (Corning, Tewksbury, MA, USA) containing serum-free medium supplemented with 2 % B-27 (ThermoFisher Scientific, Rockford, IL, USA), 20 ng/mL recombinant epidermal growth factor (EGF) and fibroblast growth factor (FGF) (Corning), and penicillin/streptomycin. After 14 days of incubation at 37 °C in a 5 % CO_2_ humidified environment, the number and size of spheroid colonies were assessed using an inverted phase-contrast microscope.

### 2.10 Transwell migration and invasion assays

As detailed in our previous study [23], migration and invasion assays were conducted. For the migration assay, 2 × 10^5^ control or MAOB-manipulated RCC cells were seeded in uncoated top chambers (8-μm pore size; Corning). In the invasion assay, 4 × 10^4^ cells were seeded in top chambers coated with Matrigel. Serum-free medium was added to the top chambers, while the lower chambers contained 10 % serum as a chemoattractant. After 24 h for migration and 48 h for invasion, cells were fixed with methanol, stained with 0.5 % crystal violet, and quantified by counting cells in three fields under 100 × magnification.

### 2.11 Flow cytometric analysis of oxidative stress

RCC cells manipulated for MAOB were seeded in a 24-well plate and treated with or without MAOB inhibitors or antioxidants for 24 h. Oxidative stress was then detected by staining cells with 5 μM DCFDA in complete medium for 30 min. After washing with PBS, ROS levels in DCFDA-preloaded cells were measured on a CytoFLEX flow cytometer (Beckman Coulter Life Sciences, Indianapolis, IN, USA).

### 2.12 Measurement of hydrogen peroxide (H_2_O_2_) using PO-1 fluorescent probe

RCC cells transfected with either MAOB-overexpression plasmid or empty vector (EV) were seeded in six-well plates and treated with H_2_O_2_, MAOB inhibitors, or antioxidants for 24 h. Following treatment, cells were incubated with 5 μM PO-1 dye in PBS for 30 min at 37 °C. After washing twice with PBS, intracellular H_2_O_2_ levels were quantified by flow cytometry.

### 2.13 Measurement of H_2_O_2_ using HyPer7.2 biosensor

RCC cells transfected with either MAOB-overexpression or EV constructs were further transfected with HyPer7.2-NLS or HyPer7.2-NES plasmids (Addgene, Watertown, MA, USA) using Lipofectamine 3000 according to the manufacturer's instructions in six-well plates. After 24 h, cells from each well were seeded into black-walled, clear-bottom 96-well plates. Cells were then treated with 200 μM H_2_O_2_ (for the EV group) or with NAC or selegiline (for MAOB-overexpressing cells). Following an additional 24-h incubation, HyPer7.2 fluorescence was measured using a multimode microplate reader. The biosensor was sequentially excited at 420 nm and 490 nm, and emission was collected at 535 nm. The fluorescence ratio (490/420 nm) was calculated to quantify intracellular H_2_O_2_ levels.

### 2.14 8-OHdG levels measurement

An 8-OHdG ELISA kit (Coon Koon Biotech Co., Ltd., Shanghai, China) was used to measure the effects of MAOB overexpression, with or without NAC treatment (5 mM), on 8-OHdG levels. Briefly, Caki-1 cells (1 × 10^5^) were lysed after five freeze–thaw cycles, and 8-OHdG concentrations were determined using the ELISA kit according to the manufacturer's instructions, following establishment of a standard curve.

### 2.15 In vivo subcutaneous and orthotopic xenograft models

RCC xenograft models were established based on protocols from our previous study [24]. Briefly, luciferase (luc)-tagged Caki-1 cells (10^6^) expressing either MAOB or a control vector were either subcutaneously or orthotopically implanted into 6–8-week-old nonobese diabetic (NOD)-severe combined immunodeficient (SCID) mice. The tumor size and location were monitored weekly using the Xenogen IVIS-200 noninvasive bioluminescent imaging system (Xenogen, Alameda, CA, USA). In the subcutaneous xenograft model, the tumor size was also measured weekly by calipers in cubic millimeters using formula 1/2 ab^2^ (where ‘a’ is the length and ‘b’ is the width). After 9 weeks, mice were sacrificed, and tumor specimens were collected, photographed, sectioned, and stained with hematoxylin and eosin (H&E) for a histopathological analysis. In the orthotopic xenograft model, mice were sacrificed after 6 weeks, and luciferase activity in excised organs was measured using the IVIS-200 system. Primary tumors were also photographed. All animal experiments were performed in accordance with protocols approved by the Institutional Animal Care and Use Committee of Taipei Medical University (approval no.: LAC2022-0312).

### 2.16 Cell-cycle analysis

To analyze the cell-cycle distribution, ccRCC cells with or without MAOB overexpression were seeded in 6-cm dishes and incubated for 24 h. After incubation, cells were harvested and fixed overnight at −20 °C in ice-cold 70 % ethanol. Fixed cells were stained with 10 μg/mL propidium iodide (PI) in the presence of 100 μg/mL RNase A and 0.1 % Triton X-100 at 37 °C for 1 h. The DNA content was measured using a CytoFLEX flow cytometer, and proportions of cells in the G_0_-G_1_, S, and G_2_-M phases were calculated using CytExpert 2.0.0 software (Beckman Coulter).

### 2.17 Terminal deoxynucleotidyl transferase dUTP nick end labeling (TUNEL) apoptosis assay

An APO-BrdU TUNEL assay kit (Enzo Lifesciences, Farmingdale, NY, USA) was utilized to label fragmented DNA during apoptosis, following the manufacturer's protocol. In brief, ccRCC cells, with or without MAOB overexpression, were seeded in 6-cm dishes and treated with various MAOB inhibitors. After 24 h of treatment, cells were harvested, fixed with 4 % paraformaldehyde, and permeabilized with ice-cold 70 % ethanol. Following ethanol removal, DNA fragments at the terminal ends of apoptotic cells were labeled with Br-2′-deoxyuridine 5′-triphosphate (dUTP) using the terminal deoxynucleotidyl transferase (TdT) enzyme. Signal detection was performed using a fluorescein-labeled anti-BrdU antibody, and measurements were obtained using either a CytoFLEX flow cytometer (Beckman Coulter) or a fluorescence microscope (Zeiss Axioplan, Zeiss, Oberkochen, Germany).

### 2.18 Mitochondrial membrane potential (MMP) measurement

Breakdown of the MMP was assessed using JC-1, which allows detection of changes in the MMP. For this purpose, ccRCC cells with or without MAOB overexpression were incubated for 24 h with complete medium following staining with JC-1 (10 μg/mL) for 10 min in the dark. Spare dye was removed by PBS washing, and cell-associated fluorescence was measured with a CytoFLEX flow cytometer or fluorescence microscopy.

### 2.19 Extraction of RNA and reverse-transcription quantitative polymerase chain reaction (RT-qPCR)

Total RNA was extracted from RCC cells using the TRIzol reagent (Invitrogen Life Technologies, Carlsbad, CA, USA) and subsequently reverse-transcribed into complementary (c)DNA using the iScript™ cDNA Synthesis Kit (Bio-Rad). Gene amplification was performed with OmicsGreen 5 × qPCR Master Mix on the StepOnePlus™ Real-Time PCR System (Applied Biosystems, Carlsbad, CA, USA). Glyceraldehyde-3-phosphate dehydrogenase GAPDH served as the internal control. The primer sequences are shown in Table S3.

### 2.20 Pathway and RNA sequencing (RNA-Seq) analysis

To identify potential upstream regulators of MAOB-mediated signaling in ccRCC, MAOB-positively associated genes were analyzed using an Ingenuity Pathway Analysis (IPA, Qiagen) for upstream regulator prediction. RNA-Seq was performed on MAOB-overexpressing and control Caki-1 cells by Genomics BioSci & Tech. (New Taipei City, Taiwan). Differentially expressed genes (DEGs) were identified, with upregulated genes (fold change (FC) > 2) and downregulated genes (FC < 0.5) visualized in a heatmap. Additionally, a gene set enrichment analysis (GSEA) was conducted to explore associations of MAOB-regulated genes with p53 and HNF1A target pathways.

### 2.21 P53 luciferase reporter assay

ccRCC cells, with or without MAOB overexpression, were treated with specified inhibitors and co-transfected for 24 h using Lipofectamine 3000. Transfection included the pGL4.38 vector containing two p53 response elements (Promega, Madison, WI, USA) and a control Renilla vector (Promega). Afterward, cell lysates were collected, and both firefly and Renilla luciferase activities were quantified using a Dual-Luciferase Reporter Assay Kit (Promega) and measured with a SpectraMax L luminescence microplate reader (Molecular Devices, San Jose, CA, USA). Renilla luciferase activity served as an internal control. The relative luciferase activity was determined using the formula: Relative luciferase activity (fold) = (luciferase activity of the MAOB-overexpression sample)/(luciferase activity of the control sample).

### 2.22 Assessment of lipid peroxidation by Liperfluo staining

Lipid peroxidation was assessed using Liperfluo (Dojindo Molecular Technologies, Rockville, MD, USA). ccRCC cells, with or without MAOB overexpression, were stained with 10 μM Liperfluo for 30 mi at 37 °C, harvested via trypsinization, and immediately analyzed by flow cytometry. The fluorescence intensity was measured in the FITC channel.

### 2.23 Chromatin immunoprecipitation (ChIP) assay

ChIP assays were performed using Caki-1 cells (10^6^) according to the manufacturer's protocol (EZ-ChIP™, cat. no. 17–295; Millipore). One microgram of anti-p53 antibody was cross-linked with protein A/G magnetic beads and used to immunoprecipitate protein-DNA complexes overnight. DNA fragments associated with p53 were eluted using 1 % SDS, 0.05 M EDTA, and 0.05 M Tris-HCl (pH 8.0), followed by reverse cross-linking at 65 °C for 2 h. Mouse immunoglobulin G (IgG) served as the negative control. Predicted p53-binding sites within the MAOB promoter region were identified using the JASPAR database (http://jaspar.genereg.net/). Enrichment of p53-bound DNA was assessed by a qPCR using the following specific primers:

forward: 5′-GCCAGGTGAGGTAGGATGAA-3′ and reverse: 5′-GGGTTAGAAGGAGCAAGAGGT-3’.

### 2.24 Statistical analysis

Results of the in vitro, in vivo, and IHC studies are expressed as the mean ± standard deviation (SD). Comparisons between two groups were conducted using Student's *t*-test. For comparisons involving three groups, a one-way analysis of variance (ANOVA) followed by Tukey's post hoc test was conducted to compare each group. Statistical analyses of clinical data from TCGA and CPTAC were described above.

## 3 Results

### 3.1 MAOB expression is downregulated in ccRCC specimens and shows negative correlations with tumor progression and a poor prognosis

**Fig. 1:**
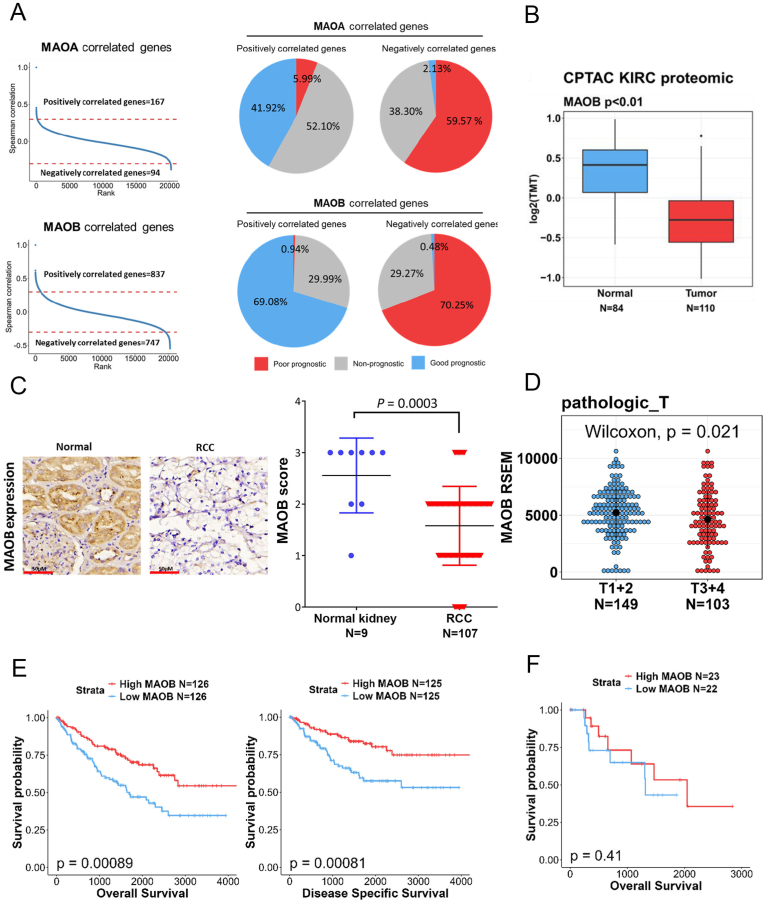
MAOB is downregulated in ccRCC tissues and inversely correlated with tumor size and a poor prognosis. **A** The graphs display the correlation ranks of MAOA- and MAOB-associated genes, with red dashed lines representing the threshold for defining correlated candidates. Genes with Spearman correlation coefficients of >0.3 or < -0.3 were respectively considered to be correlated with MAOA or MAOB expression (left panel). The pie charts show the proportions of MAOA- or MAOB-associated genes with their prognostic value. A Cox regression analysis of TCGA-KIRC patients classified genes as poor prognostic (hazard ratio (HR) > 1, *p* < 0.05), good prognostic (HR < 1, *p* < 0.05), or non-prognostic if they did not meet these criteria (right panel). **B** Box plot indicating that MAOB protein levels decreased in tumor tissues compared to normal tissues. The ccRCC dataset was retrieved from the CPTAC database. The Wilcoxon rank-sum test was performed to evaluate statistical significance. **C** MAOB protein expressions in RCC and normal tissues were analyzed by IHC staining. The left panel displays representative MAOB IHC staining images, while the right panel shows IHC expression scores comparing RCC to normal tissues. Scale bars in the left panel represent 50 μM. A *t*-test was performed to evaluate statistical significance. **D** MAOB expression levels in ccRCC samples from TCGA-KIRC were analyzed based on pathologic T stages. **E, F** Kaplan-Meier survival curves for patients with ccRCC (**E**) and papillary (p)RCC (**F**) are shown, stratified by high or low MAOB expression. The *p* value indicates the statistical comparison between these groups, with a log-rank test *p* value of <0.05 considered significant. ccRCC and pRCC datasets were obtained from TCGA.

Since the role of MAOs in RCC remains unknown, we first explored the prognostic value of both MAOA and MAOB in ccRCC using TCGA-KIRC cohort. A correlation analysis of MAO expressions with genes in ccRCC patients identified 261 MAOA-associated genes, including 167 positively correlated genes (with Spearman's correlation coefficient of ≥0.3) and 94 negatively correlated genes (with Spearman's correlation coefficient of ≤ -0.3). Similarly, 1584 MAOB-associated genes were identified, with 837 showing a positive correlation and 747 showing a negative correlation (Fig. 1A, left panel). To further evaluate the prognostic significance of MAO-correlated genes, we conducted a univariate Cox proportional hazard analysis using TCGA-KIRC data. Genes with an HR of >1 were considered poor prognostic markers, while those with an HR of <1 indicated a better prognosis. Among MAOA-correlated genes, 41.92 % of positively correlated genes were linked to a better prognosis, while 59.57 % of negatively correlated genes indicated poorer outcomes. For MAOB, 69.08 % of positively correlated genes were associated with a better prognosis, whereas 70.25 % of negatively correlated genes indicated a poor prognosis (Fig. 1A, right panel). Therefore, MAOB-associated genes showed stronger clinical relevance in ccRCC, also suggesting a potential tumor-suppressive role for MAOB in RCC. To further validate the clinical relevance of MAOB, we analyzed its expression in RCC and paired normal kidney tissues using the GSE167093 dataset, revealing significantly lower MAOB transcript levels in RCC (Fig. S1). Similarly, proteomic data from the CPTAC database confirmed reduced MAOB protein levels in ccRCC compared to normal tissues (Fig. 1B). IHC staining of a TMA further validated significantly lower MAOB protein expression in RCC tissues (Fig. S2 and 1C). Additionally, Chi-squared and Wilcoxon rank sum tests in TCGA-KIRC cohort demonstrated a negative correlation between MAOB expression and tumor sizes (Table 1, Fig. 1D). Unlike in ccRCC, MAOB expression showed no correlation with tumor sizes in pRCC patients from TCGA dataset (Table S4). Results of a Kaplan-Meier analysis further indicated that higher MAOB expression was associated with better OS and DSS in ccRCC patients (Fig. 1E) but not in pRCC patients (Fig. 1F). Additionally, univariate and multivariate Cox regression analyses identified MAOB expression as an independent prognostic factor for OS in ccRCC (Table 2). Collectively, these clinical findings suggested that MAOB is a valuable prognostic marker in RCC, especially ccRCC, and may play a crucial role in regulating tumor growth.

**Table 1:** Demographics and clinical Characteristics in 252 patients with clear cell renal carcinoma(ccRCC).

Subject characteristics	N 252	MAOB expression, N (%)		*P* value
		High 126(50)	Low 126(50)	
**Gender**				
Male	153	68(44.4)	85(55.6)	**0.039**
Female	99	58(58.6)	41(41.4)	
**Pathological stage**				
Stage1+2	137	76(55.5)	61(44.5)	0.076
Stage3+4	115	50(43.5)	65(56.5)	
**Pathologic T**				
T1+2	149	84(56.4)	65(43.6)	**0.002**
T3+4	103	42(40.8)	61(59.2)	
**Pathologic N**				
N0	236	119(50.4)	117(49.6)	0.796
N1	16	7(43.8)	9(56.2)	
**Pathologic M**				
M0	211	107(50.7)	104(49.3)	0.732
M1	41	19(46.3)	22(53.7)

**Table 2:** Univariate and multivariate overall survival analyses of MAOB and clinic pathological parameters in patients with clear cell renal cell carcinoma.

Variable	HR	Univariate	P value
		95 % C.I	
MAOB	0.50	0.33–0.76	**0.001**
Stage 1 + 2 VS. 3 + 4	3.49	2.27–5.38	**<0.001**
Pathologic T1+2 VS. 3 + 4	3.09	2.04–4.68	**<0.001**
Pathologic N0 VS. N1	3.42	1.82–6.46	**<0.001**
Pathologic M0 VS. M1	4.06	2.63–6.27	**<0.001**

Variable	HR	Multivariate	P value
		95 % C.I	
MAOB	0.51	0.33–0.78	**0.002**
Stage 1 + 2 VS. 3 + 4	1.49	0.60–3.71	0.394
Pathologic T1+2 VS. 3 + 4	1.45	0.64–3.27	0.369
Pathologic N0 VS. N1	2.04	1.05–3.95	**0.035**
Pathologic M0 VS. M1	2.75	1.66–4.55	<0.001

### 3.2 Enzymatically active MAOB suppresses aggressive behaviors of ccRCC cells via inducing ROS production

**Fig. 2:**
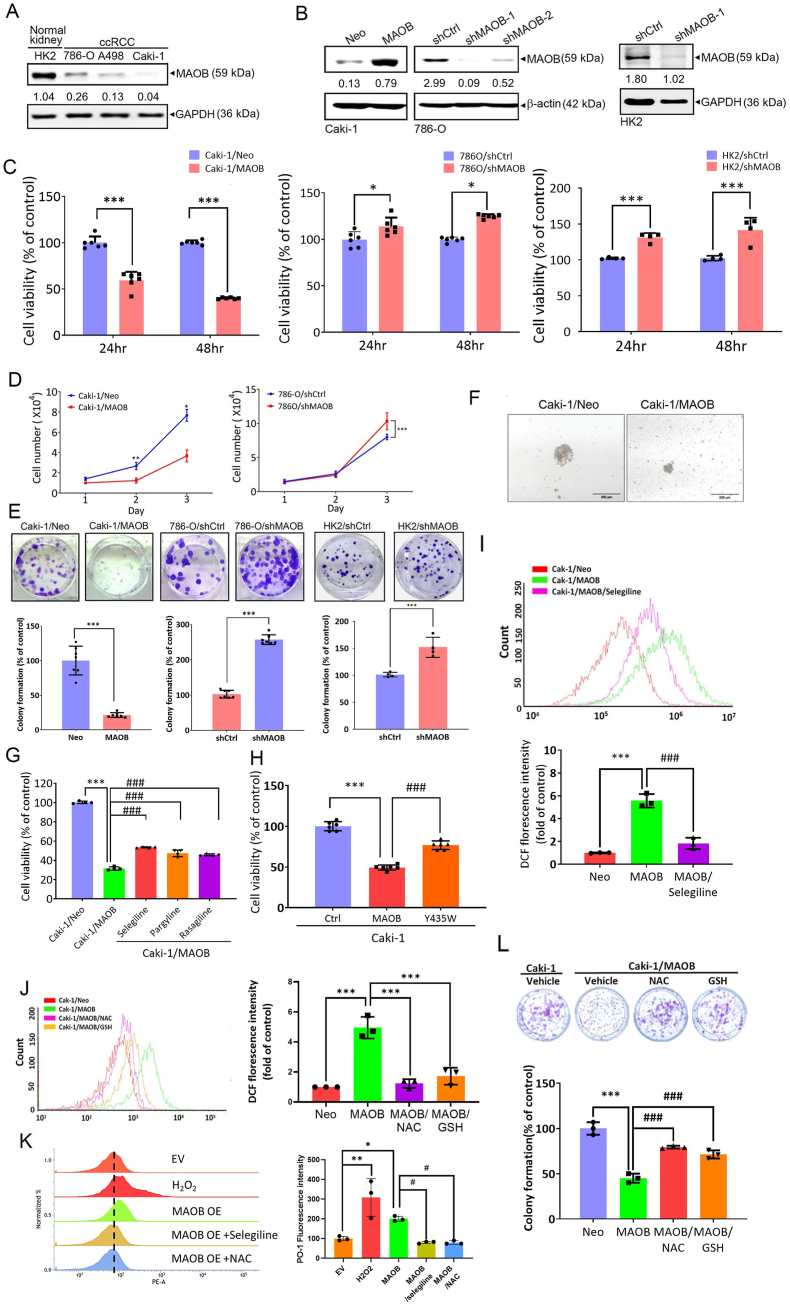
The enzymatic activity of MAOB is pivotal in suppressing growth of ccRCC cells via inducing ROS production. **A** MAOB protein levels in renal proximal tubule epithelial cells (HK2) and ccRCC cell lines were estimated by Western blotting, with GAPDH as an internal loading control. **B** MAOB was overexpressed in Caki-1 cells and knocked down in 786-O and HK2 cells, as determined by Western blotting. **C–F** MAOB expression was negatively correlated with ccRCC cell viability (Caki-1, *n* = 6; 786-O, *n* = 6; HK2, *n* = 4) (**C**), proliferation (*n* = 3) (**D**), colony formation (Caki-1, *n* = 7; 786-O, *n = *7; HK2, *n* = 4) (**E**), and sphere formation (**F**). Data in (C–E) are presented as the mean ± standard deviation (SD). ∗*p* < 0.05, ∗∗*p* < 0.01, ∗∗∗*p* < 0.001. **G** Viability of Caki-1/Neo and Caki-1/MAOB cells after treatment with or without 10 μM of various MAOB inhibitors (selegiline, pargyline, or rasagiline) for 48 h (*n* = 4). **H** Viability of Caki-1 cells after transduction with wild-type MAOB, MAOB/Y435W, or a control vector (*n* = 6). **I, J** Oxidative stress were measured using a DCFDA fluorescent probe and flow cytometry in Caki-1/Neo and Caki-1/MAOB cells treated with or without 10 μM selegiline (*n* = 3) (**I**) or 5 mM of the antioxidant reagents NAC and GSH (*n* = 3) (**J**). **K** Intracellular H_2_O_2_ levels were quantified using the PO-1 fluorescent probe followed by flow cytometric analysis in Caki-1/EV and Caki-1/MAOB cells, with or without treatment with 10 μM selegiline or 5 mM NAC (*n* = 3). Exogenous treatment of 200 μM H_2_O_2_ was used as a positive control. **L** Colony-forming ability was evaluated in Caki-1/Neo and Caki-1/MAOB cells treated with or without NAC and GSH. ∗∗*p* < 0.01, ∗∗∗*p* < 0.001 compared to the vector control group (*n* = 3). ^#^*p* < 0.05, ^##^*p* < 0.01, ^###^*p* < 0.001 compared to the MAOB-overexpressing group. For comparisons between two groups, a *t*-test was performed. For comparisons involving more than two groups, ANOVA followed by Tukey's post hoc test was used.

Based on clinical observations, we next evaluated the effect of MAOB expression on the growth of ccRCC cells. We first evaluated the basal expression level of MAOB in ccRCC cells (786-O, A498, and Caki-1) and normal kidney tubular epithelial cells (HK2). These ccRCC cell lines were selected to represent distinct genetic backgrounds and tumor behaviors. Specifically, 786-O and A498 harbor VHL mutations, whereas Caki-1 retains wild-type VHL and was derived from a metastatic ccRCC lesion. Similar to clinical observations, MAOB levels were higher in normal HK2 cells than in ccRCC cells (Fig. 2A). Compared to metastatic Caki-1 ccRCC cells, primary 786-O and A498 ccRCC cells expressed higher MAOB (Fig. 2A). Lentiviral-based RNA-KD and RNA-overexpression approaches were used to establish stable KD and overexpression of MAOB in 786-O, HK2, and Caki-1 cells (Fig. 2B). We found that overexpression of MAOB in Caki-1 cells (Caki-1/MAOB) significantly attenuated cell proliferation rates determined by both CCK8 and trypan blue dye exclusion assays (Fig. 2C and D, left panel). In contrast, MAOB-KD caused opposite effects in both 786-O and HK2 cells (Fig. 2C and D, right panel). The effect of MAOB on long-term growth (8–10 days) of Caki-1, 786-O, and HK2 cells was further determined by a colony-formation assay, which showed that the clonogenicity of these cell lines respectively increased and decreased following KD and overexpression of MAOB (Fig. 2E). The role of cancer stem cells (CSCs) in cancer development and growth has gained significant attention [25]. Since sphere formation is a key characteristic of CSCs, we investigated whether MAOB overexpression could reduce the size of ccRCC spheroids using a sphere-formation assay. As shown in Fig. 2F, Caki-1 cells formed spheroids in 3D culture. MAOB overexpression reduced the spheroid size compared to the vector control group. MAOB is a mitochondrial enzyme that catalyzes oxidative deamination of amines, producing H_2_O_2_ [26]. Here, Caki-1/MAOB cells were treated with the irreversible MAO inhibitor, pargyline, or the selective MAOB inhibitors, selegiline and rasagiline. All three inhibitors significantly reversed MAOB overexpression-induced suppression of cell proliferation (Fig. 2G) and colony formation (Fig. S3). To further validate these findings, we constructed a Y435W MAOB mutant, known to reduce MAOB catalytic activity [27]. Compared to wild-type MAOB, Y435W overexpression showed weaker inhibitory effects on cell proliferation (Fig. 2H) and colony formation (Fig. S4). Additionally, MAOB overexpression significantly reduced the migratory and invasive abilities of Caki-1 cells, while MAOB-KD enhanced 786-O cell motility (Fig. S5). Notably, Y435W overexpression exhibited a weaker inhibitory effect on cell motility than wild-type MAOB in Caki-1 cells (Fig. S6). These findings indicated that MAOB enzyme activity is essential for its anticancer effects in ccRCC cells. Indeed, MAOB overexpression led to increased oxidative stress in Caki-1 cells, as detected using the redox-sensitive dye, DCFDA. This effect was reversed by treatment with selegiline (Fig. 2I) and the antioxidants NAC and glutathione (GSH) (Fig. 2J). To further assess H_2_O_2_ production, the H_2_O_2_-sensitive dye PO-1 was used. MAOB overexpression significantly increased intracellular H_2_O_2_ levels, which were reversed by selegiline or NAC treatment (Fig. 2K). Consistently, transfection of RCC cells with the H_2_O_2_-sensitive biosensor HyPer7.2 revealed elevated cytoplasmic and nuclear H_2_O_2_ signals in MAOB-overexpressing cells, while NAC or selegiline effectively attenuated these increases (Fig. S7). Functionally, MAOB-induced suppression of colony formation was significantly restored by the antioxidants (Fig. 2L), suggesting that the inhibitory effect of MAOB on aggressive behaviors in ccRCC cells is linked to ROS production.

### 3.3 MAOB suppresses tumorigenicity and metastasis of ccRCC cells in subcutaneous and orthotopic xenograft models

**Fig. 3:**
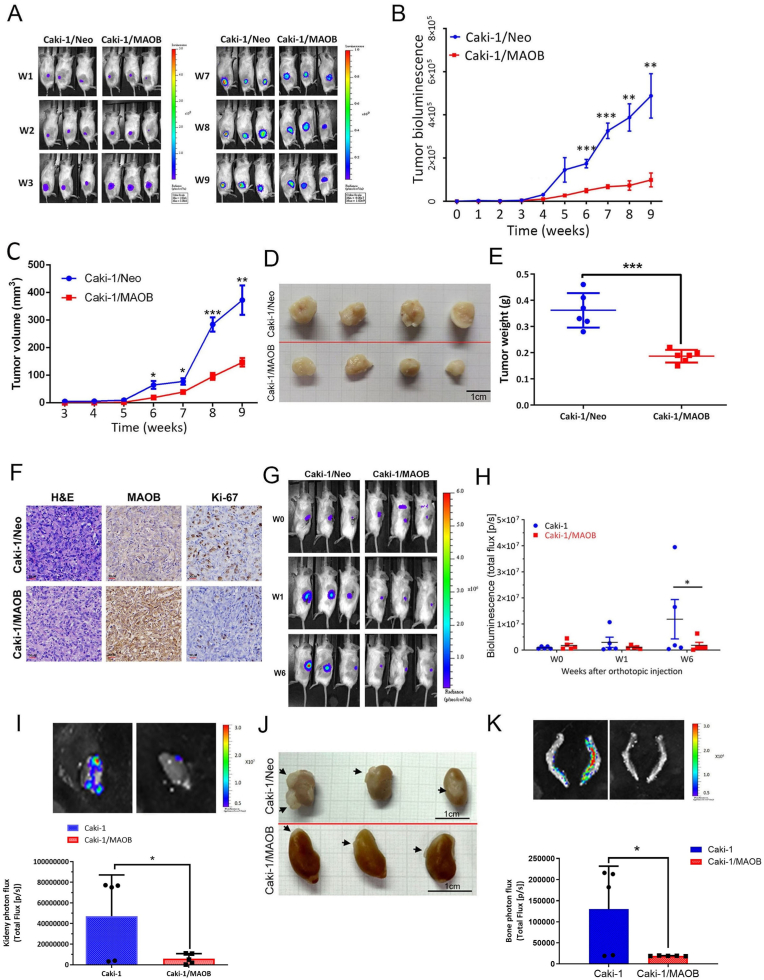
MAOB expression suppresses tumor growth and metastasis of ccRCC cells in subcutaneous and orthotopic xenograft models. **A, B** Luciferase (luc)-tagged Caki-1/Neo (*n* = 6) or Caki-1/MAOB cells (*n* = 6) were subcutaneously injected into NOD/SCID mice. Representative bioluminescence images (**A**) and quantification of Xenogen signal intensity (**B**) are shown. ∗∗*p* < 0.01, ∗∗∗*p* < 0.001 vs. the control. **C** Tumor volumes were measured with calipers in mice implanted with Caki-1/Neo or Caki-1/MAOB cells. ∗*p* < 0.05, ∗∗*p* < 0.01, ∗∗∗*p* < 0.001 vs. the control. **D, E** Gross tumor appearance (**D**) and average tumor weight (**E**) at week 9. Scale bar, 1 cm ∗∗∗*p* < 0.001 vs. the control. **F** IHC staining of MAOB and Ki-67 in Caki-1 xenografts with hematoxylin counterstaining. Scale bar, 30 μm. **G, H** NOD/SCID mice were orthotopically injected with luc-tagged Caki-1/Neo (*n* = 5) or Caki-1/MAOB (*n* = 5) cells. Whole-body bioluminescence imaging was performed weekly for 6 weeks (**G**), with signal quantification in **H**. ∗*p* < 0.05 vs. the control. **I** IVIS imaging of tumor-bearing kidneys at the study's end, showing bioluminescent signals and the mean intensity per group. ∗*p* < 0.05 vs. the control. **J** Gross appearance of orthotopic tumors (black arrow) in the kidney. **K** Ex vivo bioluminescence of metastatic bones (upper panel) and quantification of bioluminescent signals (lower panel) with mean values per group. ∗*p* < 0.05 vs. the control.

To next examine the in vivo effects of MAOB on ccRCC progression, we established a ccRCC-bearing mouse model by subcutaneously injecting luciferase (luc)-expressing Caki-1 cells (Caki-1/Neo-luc and Caki-1/MAOB-luc) into NOD-SCID mice. After 6–9 weeks, tumors from control cells (Caki-1/Neo) were larger than those from Caki-1/MAOB cells, as indicated by photon emission detection (Fig. 3A and B) and tumor volume measurements (Fig. 3C). At the end of the experiment, xenografts from the Caki-1/MAOB group had significantly lower tumor weights than those in the control group (Fig. 3D and E). Consistent with our in vitro findings, IHC staining of tumor tissues showed increased MAOB expression and a reduced Ki-67 proliferation index in Caki-1/MAOB tumors compared to Caki-1/Neo-injected tumors (Fig. 3F). The antitumor effect of MAOB was further confirmed in a ccRCC orthotopic xenograft model (Fig. 3G and H). After sacrifice, ex vivo imaging (Fig. 3I) and tumor masses (Fig. 3J) from tumor-implanted kidneys showed growth patterns consistent with the in vivo study. Additionally, ex vivo photon imaging revealed lower signal intensities in metastatic bone in the MAOB-overexpressing group compared to the control group (Fig. 3K). These data all support the crucial role of MAOB in regulating tumorigenesis and metastatic features of ccRCC cells.

### 3.4 MAOB inhibits ccRCC cell growth through ROS-triggered DNA damage, subsequently leading to G_1_ arrest, mitochondrial apoptosis, and lipid peroxidation-triggered ferroptosis

**Fig. 4:**
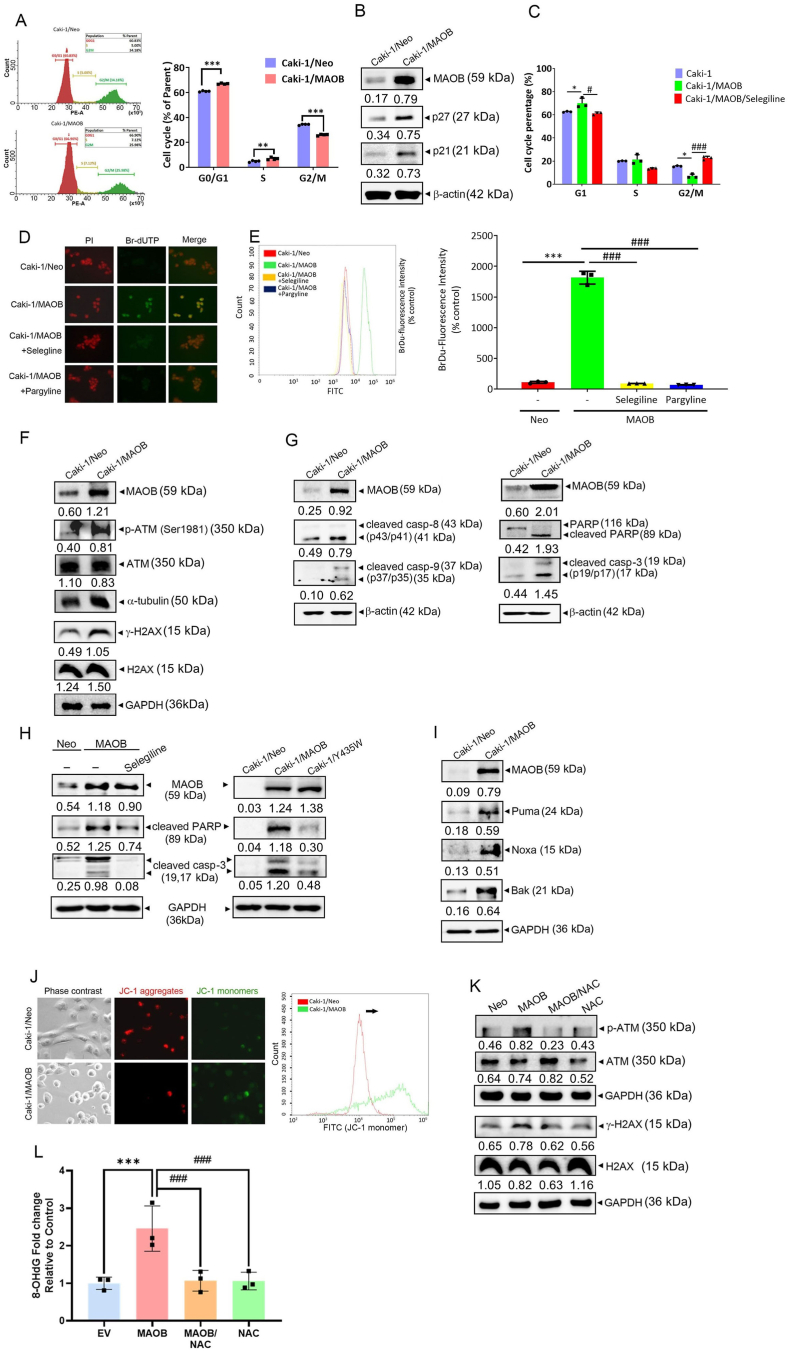
Ectopic MAOB expression induces DNA damage, G_1_ cell-cycle arrest, and mitochondrial apoptosis in ccRCC cells. **A** Flow cytometric analysis of Caki-1/Neo and Caki-1/MAOB cells stained with PI, displaying the cell-cycle distribution (left panel) and percentages of cells in the G_0_/G_1_, S, and G_2_/M phases (right panel). ∗*p* < 0.05, ∗∗∗*p* < 0.001 vs. the control (*n* = 3). **B** Western blot analysis of MAOB, p21, and p27 protein levels in Caki-1 cells following MAOB overexpression. **C** Cell-cycle distribution in Caki-1/Neo and Caki-1/MAOB cells with or without 10 μM selegiline treatment. ∗*p* < 0.05 vs. the control; ^#^*p* < 0.05, ^###^*p* < 0.001 vs. the MAOB-overexpressing group (*n* = 3). **D** IF images of Caki-1/Neo and Caki-1/MAOB cells, with or without 10 μM selegiline or pargyline treatment. DNA fragmentation was labeled with Br-dUTP, while double-stranded DNA was counterstained with PI. **E** Flow cytometric analysis of FITC-labeled dUTP in Caki-1/Neo and Caki-1/MAOB cells with or without selegiline or pargyline treatment. Left panel: fluorescence intensity histogram. Right panel: quantification. ∗∗∗*p* < 0.001 vs. the control; ^###^*p* < 0.001 vs. the MAOB-overexpressing group (*n* = 3). **F–I** Western blot analysis of MAOB, *p*-ATM, γH2AX, cleaved caspase-3, -8, and -9, PARP, Bak, Puma, and Noxa in Caki-1 cells overexpressing wild-type MAOB or MAOB/Y435W, with or without selegiline treatment. **J** JC-1 staining analyzed by IF (left panel) and flow cytometry (right panel) showing an increased green-fluorescent monomeric form in Caki-1/MAOB cells compared to Caki-1/Neo cells. **K***p*-ATM and γH2AX levels were analyzed by Western blotting in Caki-1/MAOB cells treated with or without NAC. **L** The levels of 8-OHdG were measured in MAOB- or EV-transfected Caki-1 cells with or without NAC treatment (5 mM) (*n* = 3). For comparisons between two groups, a *t*-test was performed. For comparisons involving more than two groups, ANOVA followed by Tukey's post hoc test was used.

To investigate how MAOB regulates cell growth, we first examined whether its overexpression induced cell-cycle arrest. A flow cytometric analysis revealed an increase in the G_1_ phase cell population from 60.8 % in Caki-1/Neo cells to 66.9 % in Caki-1/MAOB cells (Fig. 4A). Additionally, the G_1_ arrest-associated proteins, p21/Cip1 and p27/Kip1, were upregulated in Caki-1/MAOB cells compared to Caki-1/Neo cells (Fig. 4B). This MAOB-induced G_1_ phase arrest was significantly reversed by selegiline treatment (Fig. 4C). Beyond cell-cycle arrest, we further examined whether MAOB overexpression induced DNA fragmentation-mediated apoptosis using an APO-BrdU TUNEL assay. IF and flow cytometric analyses (Fig. 4D and E) showed increased BrdU-stained fragmented DNA in MAOB-overexpressing cells compared to controls, an effect that was reversed by MAOB inhibitor treatment. ATM is a serine-threonine kinase activated by DNA double-strand breaks (DSBs), marked by serine (Ser) 1981 autophosphorylation, and promotes cell-cycle arrest and apoptosis [28]. Activated ATM subsequently phosphorylates the histone variant H2AX at serine 139, forming γ-H2AX [29]. Here, phosphorylation of ATM at Ser1981 and H2AX at Ser139, along with the activation of caspase-8, caspase-9, caspase-3, and its downstream substrate poly(ADP ribose) polymerase (PARP), was elevated in MAOB-overexpressing Caki-1 cells compared to the controls. (Fig. 4F and G). MAOB-induced apoptosis in ccRCC was reduced when MAOB activity was inhibited by selegiline treatment (Fig. 4H, left panel) or Y435W mutant overexpression (Fig. 4H, right panel). These results suggested that enzymatically active MAOB promotes cell-cycle arrest and apoptosis in ccRCC cells, likely due to DNA damage. Elevated ROS levels can cause irreversible DNA damage and activate apoptosis pathways, including mitochondrial apoptosis [30]. As shown in Fig. 4I, MAOB overexpression increased mitochondrial proapoptotic proteins such as Bak, Puma, and Noxa. Additionally, IF (left panel) and flow cytometric (right panel) analyses of JC-1-stained Caki-1 cells (Fig. 4J) demonstrated that MAOB overexpression led to MMP collapse. Furthermore, MAOB overexpression-induced upregulation of *p*-ATM and γ-H2AX was reversed by NAC treatment (Fig. 4K). To assess the effect of MAOB overexpression on oxidative DNA damage, 8-OHdG levels were measured. MAOB overexpression led to an increase in 8-OHdG levels, which was reversed by ROS scavenging with NAC (Fig. 4L). These findings suggested that MAOB-induced ROS upregulation may trigger DNA damage, ultimately leading to mitochondrial apoptosis in ccRCC cells. In addition to apoptosis, ROS-induced DNA damage also triggers ferroptosis, a distinct form of cell death linked to lipid peroxidation [31]. Liperfluo staining revealed a significant increase in lipid peroxidation in MAOB-overexpressing Caki-1 cells, as shown in Fig. S8, suggesting that elevated MAOB levels promoted lipid peroxidation in ccRCC. Pretreatment with GSH reduced lipid peroxidation in Caki-1/MAOB cells compared to untreated cells (Fig. S8). Additionally, we analyzed the ferroptosis-related markers, glutathione peroxidase 4 (GPX4) and heme oxygenase (HO)-1, and found that GPX4 was downregulated while HO-1 was upregulated in Caki-1/MAOB cells—effects that were reversed by selegiline treatment (Fig. S9). These findings indicated that lipid peroxidation-induced ferroptosis also contributes to MAOB-mediated cell death.

### 3.5 MAOB enhances transcriptional activity and stability of p53 through post-translational modifications (PTMs), playing a role in ccRCC growth regulation

**Fig. 5:**
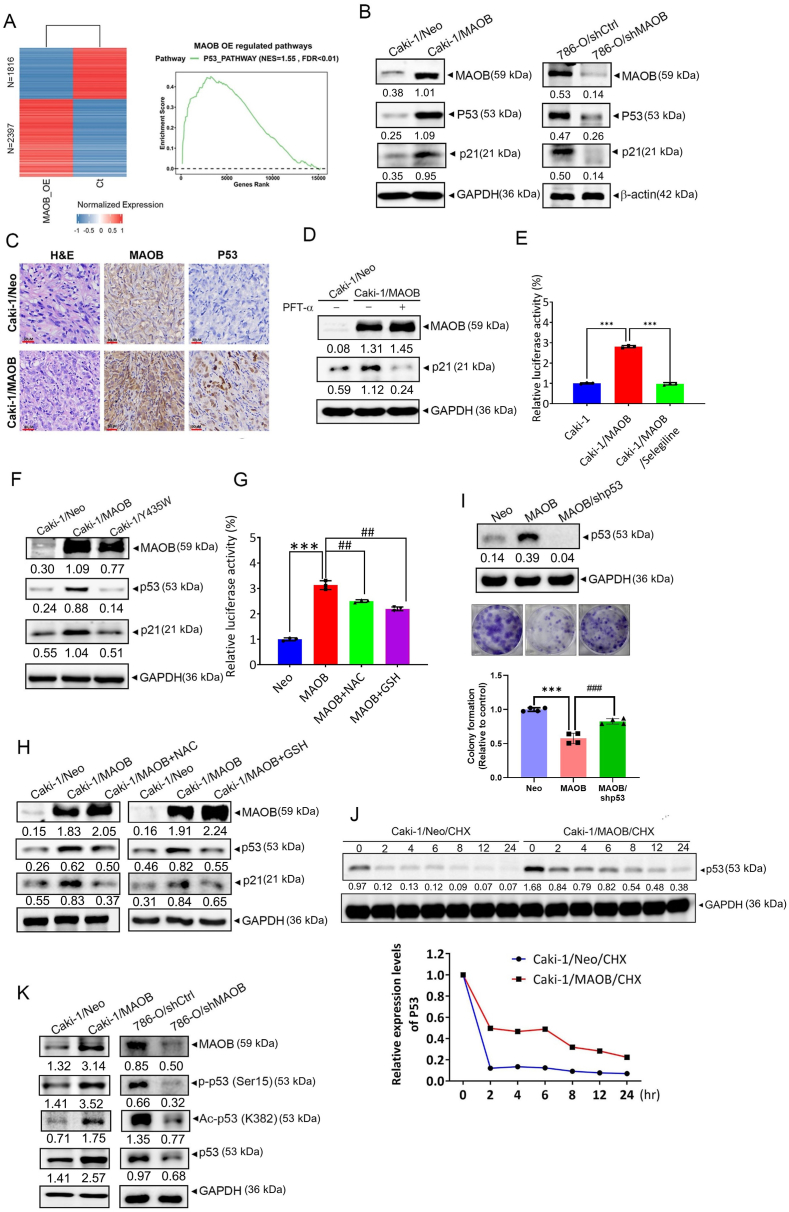
Ectopic MAOB expression suppresses ccRCC growth by enhancing p53 transcriptional activity and stability through post-translational modifications. **A** The heatmap displays genes that are downregulated (fold change (FC) < 0.5, false discovery rate (FDR) < 0.05; 1816 genes) and upregulated (FC > 2, FDR<0.05; 2397 genes) in Caki-1/MAOB cells (left panel). A gene set enrichment analysis (GSEA) revealed that the p53 signaling pathway was upregulated in the MAOB-enriched group (right panel). **B** Western blot (WB) analysis of MAOB, p53, and p21 protein levels in ccRCC cells after MAOB overexpression (left panel) and knockdown (right panel). **C** IGC staining of xenografts from Caki-1/Neo- or Caki-1/MAOB-injected mice for MAOB and p53, with hematoxylin counterstaining. Scale bar: 30 μm. **D** WB analysis of MAOB and p21 levels in MAOB-overexpressing Caki-1 cells treated with either PFT-α (10 μM) or vehicle for 24 h. **E, G** A p53 luciferase reporter was transfected into Caki-1 cells with or without MAOB overexpression and treated with or without a MAOB inhibitor (*n* = 3) (**E**) or antioxidants (*n* = 3) (**G**), as indicated. Luciferase activity was normalized to Renilla reporter activity. ∗∗∗*p* < 0.001 vs. the control; ^##^*p* < 0.01, ^###^*p* < 0.001 vs. the MAOB-overexpressing group. **F** WB analysis of MAOB, p53, and p21 in Caki-1 cells overexpressing either wild-type MAOB or the MAOB/Y435W mutant. **H** WB analysis of MAOB, p53, and p21 levels in MAOB-overexpressing Caki-1 cells treated with either antioxidants (5 mM) or vehicle for 24 h. **I** p53 shRNA was introduced into MAOB-overexpressing Caki-1 cells, followed by the measurement of p53 protein levels (upper panel) and colony-forming ability (lower panel). ∗∗∗*p* < 0.001 vs. the control; ^##^*p* < 0.01, vs. the MAOB-overexpressing group (*n* = 4). **J** Caki-1/Neo and Caki-1/MAOB cells were treated with 10 μg/mL cycloheximide at different time points, and p53 expression was assessed by WB. Quantified p53 protein levels are shown at the bottom. **K** WB analysis of phosphorylated p53 (p-p53, Ser15), acetylated p53 (Ac-p53, K382), and total p53 protein levels in ccRCC cells following MAOB overexpression (left panel) and knockdown (right panel). For comparisons between two groups, a *t*-test was performed, whereas for comparisons among more than two groups, one-way ANOVA followed by Tukey's post hoc test was applied.

RNA-Seq identified 2397 upregulated and 1816 downregulated genes in MAOB-overexpressing cells compared to the controls (Fig. 5A, left panel). A GSEA of results from RNA-Seq further revealed positive enrichment of the p53 pathways in the MAOB-overexpressing group (Fig. 5A, right panel). p53 activation in response to DNA damage leads to a rapid increase in its levels, enhancing its DNA-binding ability and transcriptional activity, which subsequently triggers genes involved in cell-cycle arrest, apoptosis, or DNA repair [32]. Herein, we observed upregulation of p53 and its downstream transcriptional targets, including p21, Bak, Puma, and Noxa, in MAOB-overexpressing Caki-1 or A498 cells, while the opposite effect was seen in MAOB-KD 786-O cells (Fig. 4I and 5B, S10). Similarly, Caki-1/MAOB xenografts exhibited higher p53 expression compared to the controls (Fig. 5C). Treatment with the p53 transcriptional activity inhibitor, pifithrin (PFT)-α, reduced MAOB-induced upregulation of p21, Puma, and Bak (Fig. 5D–S11). Moreover, a p53 reporter assay confirmed that MAOB overexpression significantly increased p53 activity in Caki-1 cells, an effect reversed by inhibitors of MAOB (Fig. 5E) and ROS (Fig. 5G). Furthermore, MAOB-induced upregulation of p53 protein levels was diminished by blocking MAOB activity (Fig. 5F) or ROS production (Fig. 5H). Collectively, these findings suggested that enzymatically active MAOB promotes ROS production, which in turn enhances p53 activity and expression in ccRCC cells. Moreover, the anti-growth effect of MAOB was significantly reversed upon p53-KD, underscoring p53's crucial role in MAOB-mediated suppression of ccRCC growth (Fig. 5I). We then examined how MAOB influences p53 expression and activity, starting with its effect on p53 protein stability. Using CHX, a protein synthesis inhibitor, we observed that p53 degradation occurred more slowly in Caki-1/MAOB cells compared to Caki-1/Neo cells (Fig. 5J), suggesting that MAOB prolongs the p53 half-life. To date, it is known that p53 transcriptional activity and stability are primarily regulated through PTMs such as phosphorylation, acetylation, methylation, ubiquitylation, and sumoylation [33]. Herein, we found that phosphorylation at Ser15 and acetylation at Lys382—the most common modifications known to enhance p53 stabilization and activation—were upregulated in MAOB-overexpressing Caki-1 cells and downregulated in MAOB-KD 786-O cells (Fig. 5K).

### 3.6 The HNF1A-53BP1 axis is essential for MAOB-induced p53 activation, which in turn positively regulates MAOB expression in ccRCC

**Fig. 6:**
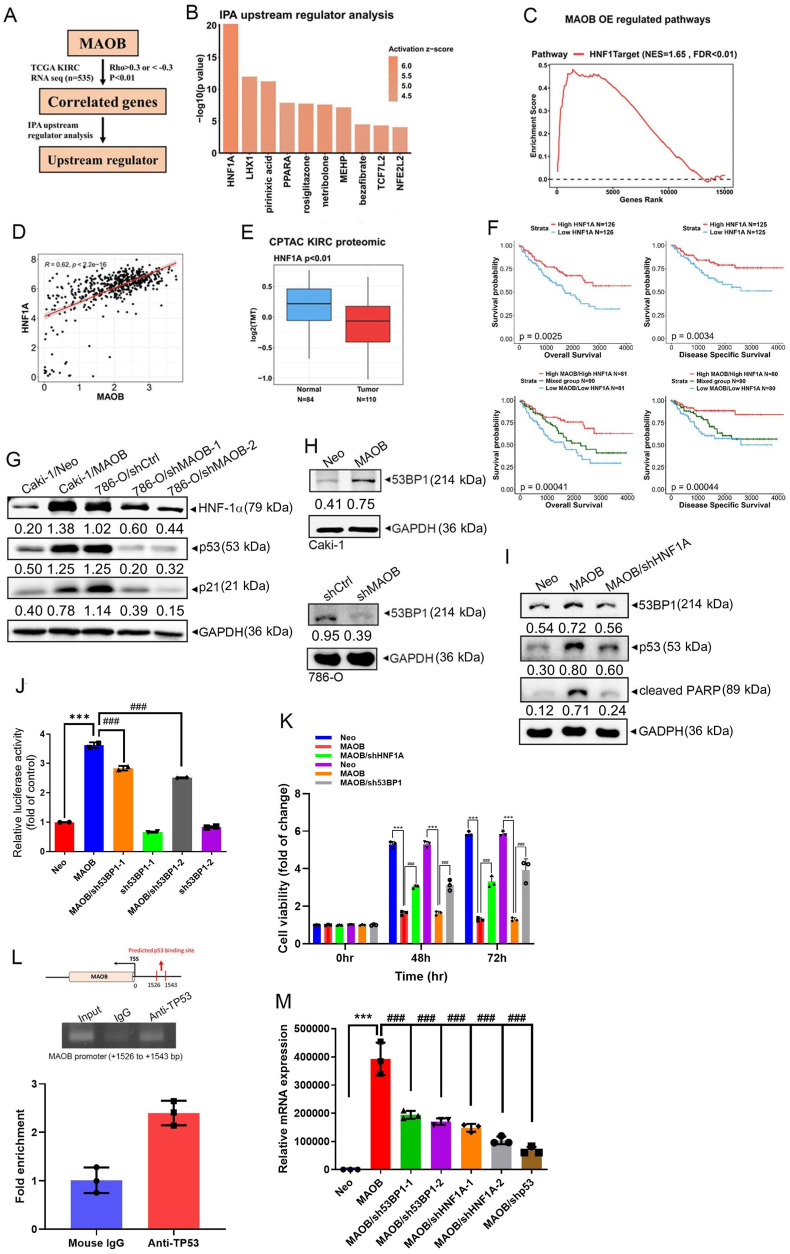
The HNF1A-53BP1 axis is essential for the MAOB-induced increase in p53 activity, and p53 activation, and it in turn, positively regulates MAOB expression in ccRCC. **A** The flowchart outlines the strategy used to identify the upstream regulator of MAOB-associated genes in TCGA-KIRC cohort. **B** The bar plot presents the top ten upstream regulators with an activation z-score of >0 and a *p* value of <0.01. **C** Gene set enrichment analysis (GSEA) based on RNA sequencing revealed that the HNF1-targeted pathway is upregulated in MAOB-enriched ccRCC cells. **D** The dot plot shows a positive correlation between MAOB and HNF1A expressions in TCGA-KIRC patients. **E** The boxplot illustrates that HNF1A protein levels were lower in tumor tissues compared to normal tissues. The ccRCC dataset was obtained from the CPTAC database. **F** Kaplan-Meier plot demonstrating that high HNF1A expression was associated with improved overall and disease-specific survival (upper panel). Moreover, patients with combined high *HNF1A* and *MAOB* expressions exhibited the best survival outcomes compared to those with low expression of either or both genes (lower panel). **G, H** Western blot analysis of HNF-1α, p53, p21 (**G**), and 53BP1 (**H**) protein levels in ccRCC cells following MAOB overexpression or knockdown. **I** HNF1A shRNA was introduced into MAOB-overexpressing Caki-1 cells, followed by an assessment of 53BP1, p53, and cleaved PARP levels. **J, K** 53BP1 or HNF1A shRNAs were introduced into MAOB-overexpressing Caki-1 cells, and p53 activity (*n* = 2) (**J**) and cell viability (*n* = 3) (**K**) were evaluated. ∗∗∗*p* < 0.001 vs. the control; ^##^*p* < 0.01, ^###^*p* < 0.001 vs. the MAOB-overexpressing group. **L** Upper panel: Representative plots illustrating the predicted p53-binding region within the MAOB promoter, located 1526–1543 bp upstream of the transcriptional start site (TSS). Middle panel: ChIP-PCR demonstrated that p53 binds to the predicted binding sites within the MAOB promoter region. Input DNA represents one-tenth of the starting material, and mouse IgG was used as a negative control. Lower panel: The bar plot shows quantitative results from the PCR. Relative fold enrichment was calculated by normalizing the signal from anti-p53 (TP53) to that from mouse IgG. ∗*p* < 0.05 vs. the IgG control (*n* = 3). **M** An RT-qPCR was performed to evaluate MAOB mRNA levels in Caki-1 and Caki-1/MAOB cells, with or without knockdown of 53BP1, HNF1A, or p53. ∗∗∗*p* < 0.001 vs. the control; ^##^*p* < 0.01, ^###^*p* < 0.001 vs. the MAOB-overexpressing group (*n* = 3). For comparisons between two groups, a *t*-test was performed, whereas for comparisons among more than two groups, one-way ANOVA followed by Tukey's post hoc test was applied.

In addition to the MAOB-induced PTMs of p53, we further also explored molecular mechanisms involving MAOB by analyzing MAOB-correlated genes in ccRCC patients from TCGA. Using an Ingenuity Pathway Analysis (IPA), we identified hepatocyte nuclear factor 1 homeobox A (*HNF1A*) as the most significant upstream regulator (Fig. 6A and B). Consistently, a GSEA of our RNA-Seq data revealed positive enrichment of the HNF1A-targeted pathway in MAOB-overexpressing ccRCC cells (Fig. 6C). Clinically, MAOB and HNF1A transcripts were highly positively correlated in ccRCC tissues (Fig. 6D). Similar to MAOB, HNF1A-encoded HNF-1α protein levels were significantly lower in ccRCC patients compared to normal tissues (Fig. 6E). Additionally, higher HNF1A expression was associated with smaller tumor sizes (Table S5) and improved OS and DSS (Fig. 6F, upper panel), and served as an independent prognostic factor for OS in ccRCC patients (Table S6). Patients who exhibited high expressions of both *MAOB* and *HNF1A* had the best OS and DSS compared to those with other expression patterns of both genes (Fig. 6F, lower panel).

Notably, MAOB overexpression upregulated HNF-1α, p53, and p21, whereas MAOB-KD reduced their expressions in Caki-1 and 786-O cells (Fig. 6G). Collectively, these findings suggested that HNF1A may act as a co-regulator of the tumor-suppressive effects of MAOB. Previous studies showed that HNF1A regulates apoptosis and cell-cycle progression in pancreatic cancer by transcriptionally activating p53-binding protein 1 (53BP1), which subsequently enhances p53 activity and stability [[34], [35], [36], [37]]. Herein, we observed that 53BP1 expression was upregulated in Caki-1/MAOB cells and downregulated in 786-O/shMAOB cells compared to their respective controls (Fig. 6H). Moreover, HNF1A-KD reversed MAOB-induced upregulation of 53BP1 and p53 (Fig. 6I), while 53BP1-KD suppressed the MAOB-induced increase in p53 activity (Fig. 6J). Furthermore, MAOB overexpression-mediated PARP cleavage and growth inhibition were significantly rescued by HNF1A-KD or 53BP1-KD (Fig. 6I–K). Collectively, these findings suggested that the HNF1A-53BP1 axis plays a critical role in MAOB-modulated p53 activity and expression, contributing to suppression of ccRCC growth. According to predictions from the JASPAR database, putative p53-binding sites were identified upstream of the MAOB transcriptional start site (TSS) (Table S7). To validate this prediction, we performed a ChIP assay and confirmed that p53 binds to the predicted site within the MAOB promoter region (Fig. 6L). Moreover, KD of HNF1A, 53BP1, or p53 reduced *MAOB* messenger (m)RNA expression in Caki-1/MAOB cells (Fig. 6M), suggesting that p53 activation induced by MAOB may exert a positive feedback regulatory effect on MAOB expression in ccRCC cells.

### 3.7 DNMT inhibitors as potential inducers of MAOB expression in ccRCC

**Fig. 7:**
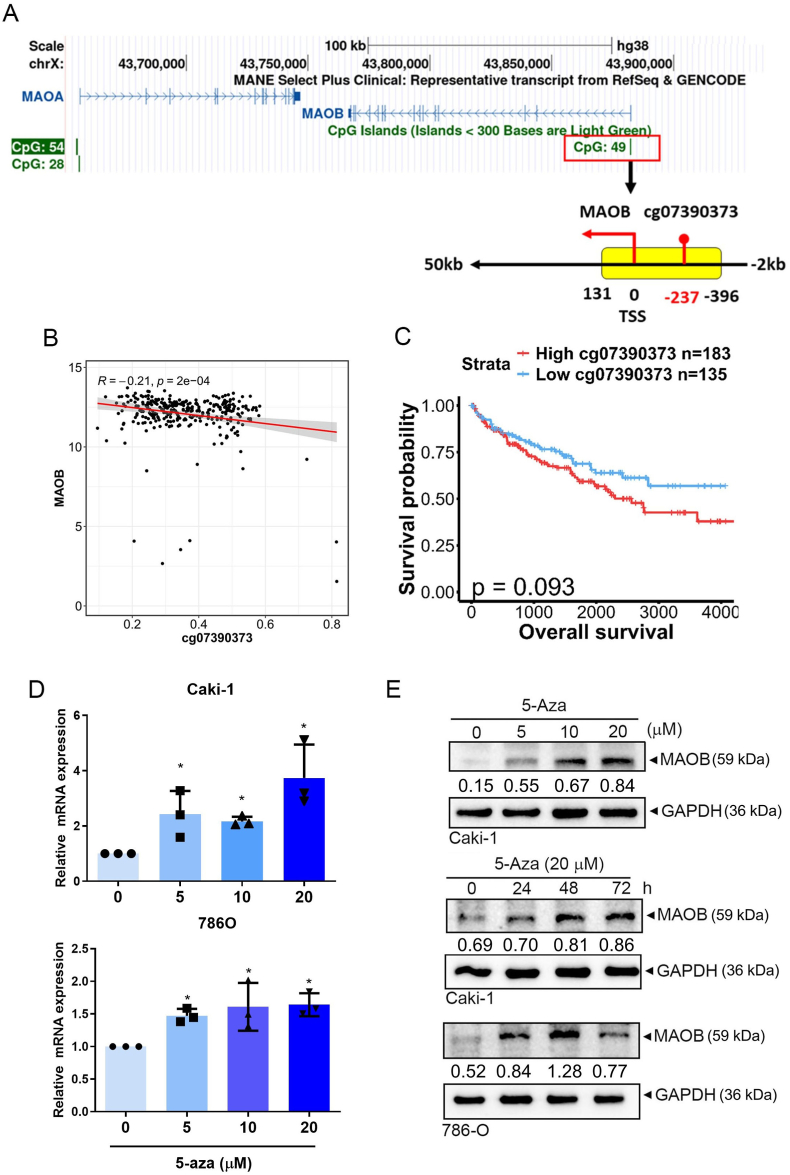
Hypomethylating agent treatment significantly increases MAOB expression in ccRCC cells by inhibiting its methylation. **A** Screenshots of the UCSC genome browser showing the location of the CpG island of MAOB. There are 49 CpG dinucleotides spanning from −396 to 131 bp using the transcription start site as the start point. The CpG probe, cg07390373, located at −237 bp is shown in the plot. **B** The dot plot illustrates a negative correlation between the methylation beta value of cg07390373 and *MAOB* gene expression. **C** The Kaplan-Meier plot indicates that high methylation of cg07390373 was associated with a trend toward poorer survival. **D, E** MAOB mRNA (**D**) and protein (**E**) levels in Caki-1 and 786-O cells were respectively assessed using an RT-qPCR (*n* = 3) and Western blot analyses, after cells were treated with the indicated concentrations of 5-Aza for 24 h or 20 μM 5-Aza for the indicated time points. ∗*p* < 0.05 vs. the control. For comparisons between two groups, a *t*-test was performed, whereas for comparisons among more than two groups, one-way ANOVA followed by Tukey's post hoc test was applied.

After elucidating the tumor-suppressive role and related mechanisms of MAOB in ccRCC, we next searched for potential inducers of MAOB. Hypermethylation of the MAOA promoter region was reported to contribute to its downregulation in cholangiocarcinomas and nasopharyngeal carcinoma. Treatment with the DNA methyltransferase (DNMT) inhibitor, 5-aza-2′-deoxycytidine (5-Aza), was shown to upregulate MAOA in both cancer types [15,38]. A computational analysis of the MAOB promoter was conducted using the UCSC genome browser (https://genome.ucsc.edu/) to identify potential CpG islands (CpGIs). One putative CpGI was identified and is depicted in Fig. 7A. The CpGI contains 49 potential CpG dinucleotides and spans the start codon for MAOB transcription. Analysis of TCGA methylation array data from ccRCC patients revealed that a methylation probe (cg07390373) located within the CpGI of the MAOB promoter exhibited a negative correlation with *MAOB* gene expression (Fig. 7B). Additionally, the high methylation intensity at this site was associated with a poorer survival trend in ccRCC patients (Fig. 7C).

Treatment of Caki-1 and 786-O cells with various concentrations of 5-Aza or 20 μM 5-Aza at indicated time points resulted in increased MAOB mRNA (Fig. 7D) and protein expressions (Fig. 7E). These findings indicated that epigenetic silencing is one of the mechanisms responsible for the low levels of MAOB expression in ccRCC and hypomethylating agents may be potential inducers of MAOB.

## 4 Discussion

Epidemiological studies identified a link between neurodegenerative disorders and either an increased or decreased risk of various cancer types [39]. MAOs, which play a crucial role in regulating neurodegenerative disorders [9], also exhibit conflicting roles in different cancers [40]. In our research, we first demonstrated that MAOB expression was downregulated in ccRCC by epigenetic methylation and further identified MAOB as a negative regulator of ccRCC malignancy. To unveil the underlying mechanism of how MAOB affects ccRCC proliferation and growth, comprehensive studies were performed, and the data indicated that MAOB suppressed ccRCC cell growth by inducing ROS-mediated DNA damage, subsequently enhancing the stability and transcriptional activity of p53, which caused G_1_ cell-cycle arrest, mitochondrial apoptosis, and lipid peroxidation-triggered ferroptosis.

Although p53 remains wild-type in approximately 87 % of carcinomas, in ccRCC, its functional activity is diminished [41], suggesting that the tumor-suppressive role of p53 in ccRCC may be compromised by unknown regulatory mechanisms. DNA damage was reported to induce PTMs of p53, leading to its activation and stabilization [33]. In our study, we observed that MAOB expression enhanced key PTMs of p53 such as Ser15 phosphorylation [28]. Phosphorylation at Ser15 is a crucial step for p53 stabilization, as it disrupts mouse double minute 2 (MDM2)-mediated inhibition and binding. Additionally, Ser15 phosphorylation enhances the interaction with the transcriptional coactivator, CREB-binding protein (CBP), promoting p53-mediated transcription. ATM-mediated phosphorylation of p53 serves as a key mechanism by which p53 responds to DNA damage [28]. Notably, in MAOB-overexpressing ccRCC, DNA damage-induced ATM activation was also observed, suggesting that the ATM-p-p53 axis is one of the critical mechanisms in MAOB-mediated reactivation of p53 in ccRCC.

CBP/p300 functions not only as a transcriptional coactivator but also as an acetyltransferase. It was reported to acetylate p53 at lysine residues K370, K372, K373, K381, K382, and K386 within its C-terminal domain (CTD), preventing interactions with MDM2 and SET, thereby stabilizing p53 and enhancing its transcriptional activity [42,43]. Cai et al. identified polybromo-1 (PBRM1) as a key reader of p53 acetylation at K382 in ccRCC. DNA damage increases both p53 levels and K382 acetylation, creating a binding signal for PBRM1 that enhances transcriptional activity. By binding to K382Ac on p53, PBRM1 may prolong p53 retention at target promoters, facilitating full activation of its downstream genes [44]. Given this role, PBRM1 is recognized as a tumor suppressor in ccRCC; however, mutations or loss of PBRM1 are frequently observed in ccRCC [45]. Our study demonstrated that MAOB-induced DNA damage also enhanced p53 K382 acetylation and upregulated PBRM1 (Fig. S12) in ccRCC cells, suggesting that PBRM1 recognition of this PTM may serve as another mechanism contributing to MAOB-mediated p53 reactivation. However, further investigation is needed to elucidate the impact of MAOB on interactions among SET, PBRM1, and p53.

Beyond the MAOB-induced PTMs of p53, an IPA of MAOB-correlated genes in ccRCC patients identified *HNF1A* as the most significant upstream regulator. HNF1A is predominantly expressed in the liver but is also found in the kidneys, intestines, and pancreas [46]. In pancreatic cancer, HNF1A was shown to transcriptionally activate 53BP1 expression, with its expression negatively correlated with oxaliplatin chemoresistance in pancreatic ductal carcinoma (PDAC) tissues and cell lines. The DNA damage response protein, 53BP1, is crucial for repairing DSBs through the nonhomologous end joining (NHEJ) pathway rather than homologous recombination (HR) [47]. Compared to NHEJ, HR-dependent DNA repair is more precise, whereas NHEJ-dependent repair often leads to genomic instability and cell death [48]. Our findings demonstrated that MAOB expression enhanced the upregulation of both HNF1A and 53BP1 in ccRCC cells. Furthermore, MAOB expression was strongly correlated with HNF1A levels in ccRCC tissues. KD of either HNF1A or 53BP1 effectively reversed MAOB-induced cell death, suggesting that the HNF1A-53BP1 axis may promote NHEJ-dependent DNA repair in response to MAOB-induced DNA damage, ultimately leading to cell death. Furthermore, 53BP1 oligomers were reported to interact with the deubiquitylase, USP28, enhancing p53-dependent transcriptional activation of its target genes [49]. Consistent with this, our study demonstrated that KD of 53BP1 reversed the MAOB-induced increase in p53 transcriptional activity, indicating that the 53BP1/USP28 axis might be critical for MAOB-mediated enhancement of p53 transcriptional regulation in ccRCC cells.

Since the antitumor effects and underlying mechanisms of MAOB in ccRCC development were thus identified, we next explored potential inducers of MAOB in ccRCC. Previous studies reported that hypermethylation of the MAOA promoter contributes to its downregulation in cholangiocarcinomas and nasopharyngeal carcinoma, while treatment with the DNMT inhibitor, 5-Aza, restores its expression in both cancers [15,38]. Similarly, we found that CpGI hypermethylation may occur in the MAOB promoter, and 5-Aza treatment increased MAOB expression in ccRCC cells, suggesting that hypomethylating agents could serve as potential MAOB inducers in ccRCC. Beyond ccRCC, MAOB was reported to be a tumor suppressor and is downregulated in head and neck squamous cell carcinoma (HNSCC) [20]. Notably, TCGA methylation array data from HNSCC patients revealed that methylation at cg07390373 within the MAOB promoter CpGI was negatively correlated with MAOB expression and was associated with poorer patient survival (Fig. S13). 5-Aza treatment also upregulated MAOB expression in SAS HNSCC cells (Fig. S14), indicating that methylation blockade may be a potential strategy for inducing MAOB in several cancers.

A limitation of this study is that while MAOB overexpression decreased JC-1 aggregates and increased JC-1 monomers, suggesting a potential reduction in MMP, JC-1–based measurements have inherent constraints [50]. This assay relies on the aggregation-dependent spectral shift of the JC-1 dye; however, its fluorescence in intact cells can be influenced not only by MMP but also by mitochondrial mass, probe concentration, and self-quenching. Therefore, future studies should include proper calibration and correction for these potential confounding factors to accurately determine the impact of MAOB on MMP.

## 5 Conclusion

**Fig. 8:**
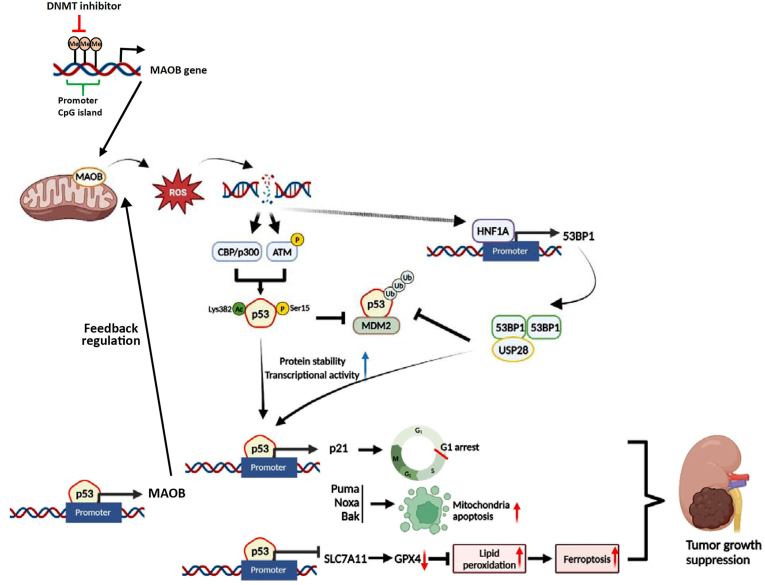
Schematic diagram depicting the impact of MAOB expression on ccRCC cell growth and its underlying mechanisms (created with BioRender.com). MAOB expression was upregulated by hypomethylating agent treatment, leading to p53 reactivation and enhanced stability through ROS-mediated DNA damage. This damage subsequently induced p53 post-translational modifications and activated the HNF1A-53BP1 axis. Upon p53 reactivation, MAOB inhibits tumor growth primarily by promoting cell-cycle arrest, mitochondrial apoptosis, and lipid peroxidation-driven ferroptosis. p53 reactivation also reinforces MAOB expression through positive feedback.

In conclusion, we demonstrated for the first time that MAOB functions as a potential tumor suppressor, inhibiting ccRCC growth. MAOB plays a crucial role in reactivating p53 and enhancing its stability by inducing ROS-mediated DNA damage, which subsequently triggers p53 PTMs and activates the HNF1A-53BP1 axis. Upon p53 reactivation, MAOB suppresses tumor growth primarily through cell-cycle arrest, mitochondrial apoptosis, and lipid peroxidation-mediated ferroptosis. In addition, we identified a novel cyclic MAOB-p53 axis that suppresses the growth of ccRCC. Our study provides valuable insight into the potential of MAOB as a novel predictor of clinical outcomes and suggests that enhancing MAOB expression or enzyme activity could be a promising therapeutic strategy for ccRCC. We identified a correlation between MAOB expression and methylation levels in ccRCC and demonstrated that hypomethylating agents serve as potential inducers of MAOB. A summary of our findings is presented in Fig. 8.

## Availability of data and materials

All data generated or analyzed during this study are included in this published article and its additional files.

## Ethics approval and consent to participate

All animal experiments were carried out in accordance with guidelines of a protocol approved by the Taipei Medical University Animal Ethics Research Board.

## CRediT authorship contribution statement

**Kuo-Hao Ho:** Data curation, Formal analysis, Methodology, Software, Validation, Writing – review & editing. **Yung-Wei Lin:** Investigation, Methodology, Software, Writing – review & editing. **Hsiang-Ching Huang:** Data curation, Investigation, Methodology, Resources, Validation, Visualization, Writing – original draft. **Feng-Ru Lai:** Investigation, Methodology, Writing – review & editing. **Yi-Chieh Yang:** Data curation, Methodology, Writing – review & editing. **Chung-Howe Lai:** Data curation, Writing – review & editing. **Yu-Ching Wen:** Data curation, Writing – review & editing. **Feng-Koo Hsieh:** Formal analysis, Writing – review & editing. **Wei-Jiunn Lee:** Data curation, Formal analysis, Investigation, Methodology, Writing – original draft, Writing – review & editing. **Ming-Hsien Chien:** Conceptualization, Data curation, Formal analysis, Funding acquisition, Methodology, Visualization, Writing – original draft, Writing – review & editing.

## Declaration of competing interest

None.
