# Supplementary material for: MAOB promotes ROS-mediated DNA damage, triggering a cyclic MAOB-HNF1A-53BP1-p53 axis that suppresses the malignancy of clear cell renal cell carcinoma
Source: Redox Biol. 2025 Nov 25;88:103945. doi: 10.1016/j.redox.2025.103945 (PMC12719070; doi:10.1016/j.redox.2025.103945)

**Supplemental Information**

**Title:**

**MAOB promotes ROS-mediated DNA damage, triggering a cyclic MAOB-HNF1A-53BP1-p53 axis that suppresses the malignancy of clear cell renal cell carcinoma**

Kuo-Hao Ho, Yung-Wei Lin, Hsiang-Ching Huang, Feng-Ru Lai, Yi-Chieh Yang, Chung-Howe Lai, Yu-Ching Wen, Feng-Koo Hsieh, Wei-Jiunn Lee, and Ming-Hsien Chien

Correspondence to: Dr. Wei-Jiunn Lee (E-mail: wjlee@tmu.edu.tw) and Dr. Ming-Hsien Chien (E-mail: mhchien1976@gmail.com)

**Supplementary Table 1.** List of antibodies utilized

| **Antibody** | **Catalog** | | **Company** | | **Application** | | **Dilution** |  |
| --- | --- | --- | --- | --- | --- | --- | --- | --- |
| MAOB  MAOB | | ab133270  AV43557 | | Abcam, Cambridge, UK  Sigma-Aldrich, USA | | WB  IHC | 1:1000  1:100 | |
| p21 | | sc-817 | | Santa Cruz Biotechnology, USA | | WB | 1:1000 | |
| p27  p53  p-p53 (Ser15)  Ac-p53 (K382)  53BP1  HNF-1α  Ki-67 | | sc-6246  sc-126  sc-51690  ab75754  sc-515841,  sc-393925  9027S | | Santa Cruz Biotechnology, USA  Santa Cruz Biotechnology, USA  Santa Cruz Biotechnology, USA  Abcam, Cambridge, UK  Santa Cruz Biotechnology, USA  Santa Cruz Biotechnology, USA  Cell Signaling Technology, USA | | WB  WB/IHC/ChIP  WB  WB  WB  WB  IHC | 1:1000  1:1000  1:1000  1:1000  1:1000  1:1000  1:100 | |
| Cleaved casp-8 | | 8592 | | Cell Signaling Technology, USA | | WB | 1:1000 | |
| Cleaved casp-9 | | 7237 | | Cell Signaling Technology, USA | | WB | 1:1000 | |
| Cleaved casp-3 | | 9664 | | Cell Signaling Technology, USA | | WB | 1:1000 | |
| Cleaved PARP | | 5625 | | Cell Signaling Technology, USA | | WB | 1:1000 | |
| Puma | | sc-374223 | | Santa Cruz Biotechnology, USA | | WB | 1:1000 | |
| Noxa | | sc-56169 | | Santa Cruz Biotechnology, USA | | WB | 1:1000 | |
| Bak | | sc-518110 | | Santa Cruz Biotechnology, USA | | WB | 1:1000 | |
| HO-1 | | sc-136960 | | Santa Cruz Biotechnology, USA | | WB | 1:1000 | |
| GPX4  p-ATM (Ser1981)  γ-H2AX (Ser139) | | sc-176570  5883  NB100-384 | | Santa Cruz Biotechnology, USA  Cell Signaling Technology, USA  Novus Biologicals, USA | | WB  WB  WB | 1:1000  1:1000  1:1000 | |
| β-actin | | 4970 | | Cell Signaling Technology, USA | | WB | 1:1000 | |
| GAPDH  α-tubulin | | 60004-1-1g  66031-1 | | Proteintech, USA  Proteintech, USA | | WB  WB | 1:1000  1:1000 | |

**Supplementary table 2.** List of shRNA target sequences

| **Gene** | **Species** | **Target sequence (5'–3')** |
| --- | --- | --- |
| MAOB shRNA-1 | human | CCCAGAATCGTATCTTGAGAT |
| MAOB shRNA-2 | human | TAGGATTGGAGACCTACAAAG |
| P53 shRNA | human | CACCATCCACTACAACTACAT |
| 53BP1 shRNA-1  53BP1 shRNA-2 | human  human | GATACTCCTTGCCTGATAATT  CCCTTGTTCAGGACAGTCTTT |
| HNF1A shRNA-1  HNF1A shRNA-2 | human  human | GTCCCTTAGTGACAGTGTCTA  GCTCCCGCAGACTATGCTCAT |

**Supplementary Table 3.** Primer sequences used in this study

| **Gene** | **Primer** | **Target sequence (5'–3')** |
| --- | --- | --- |
| MAOB | F | ATGACATGGGGCGAGAGATTC |
|  | R | GCAAGCTGCTTTGCAGATT |
| GAPDH | F | CACCATCCACTACAACTACAT |
|  | R | TTCTTACTCCTTGGAGGC CATGTA |

**Supplementary Table 4.** Demographics and clinical characteristics of 45 patients with papillary renal cell carcinoma (pRCC)


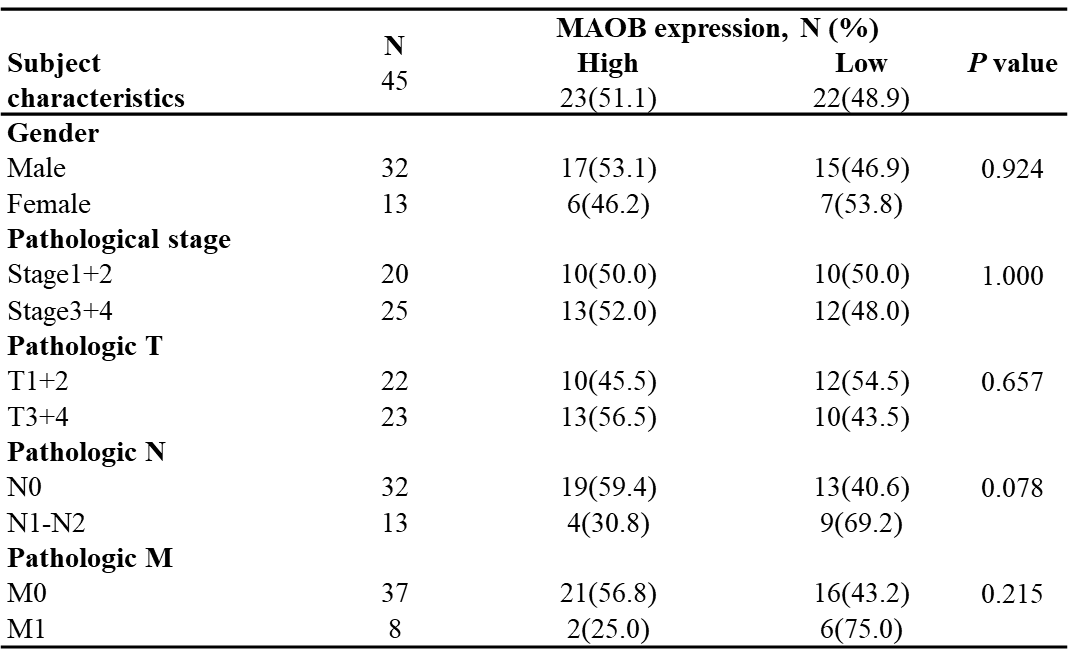


**Supplementary Table 5.** Demographic and clinical characteristics of 252 patients with clear cell renal carcinoma (ccR**
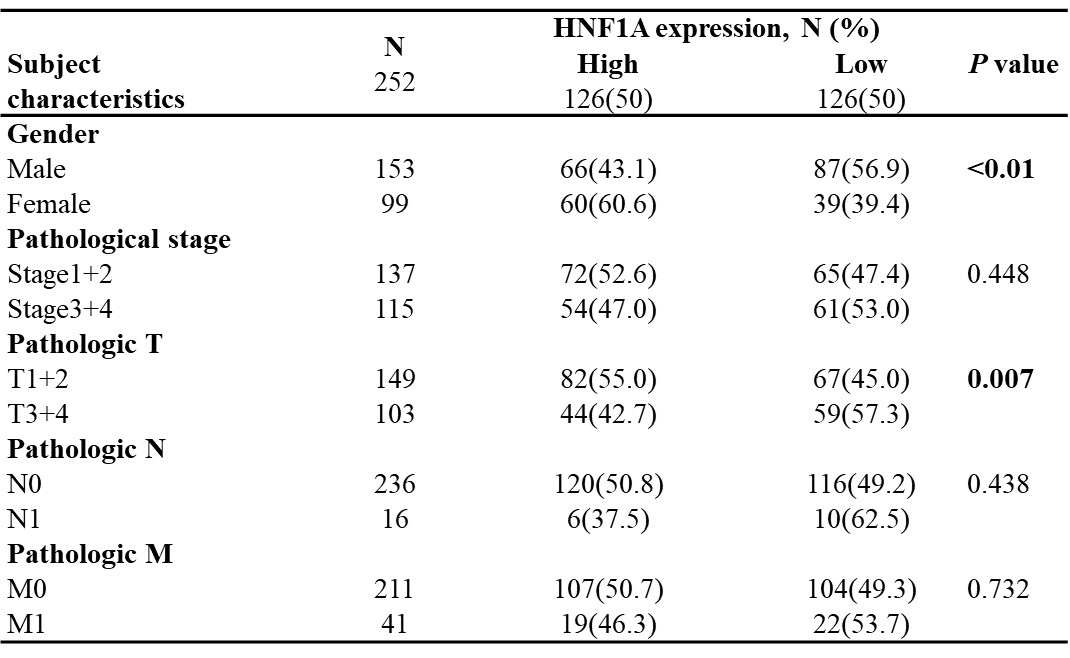
**CC)

**Statistical significance *p*<0.05 shown in bold.**

**Supplementary Table 6.** Univariate and multivariate overall survival analyses of HNF1A and clinic pathological parameters in patients with clear cell renal cell carcinoma


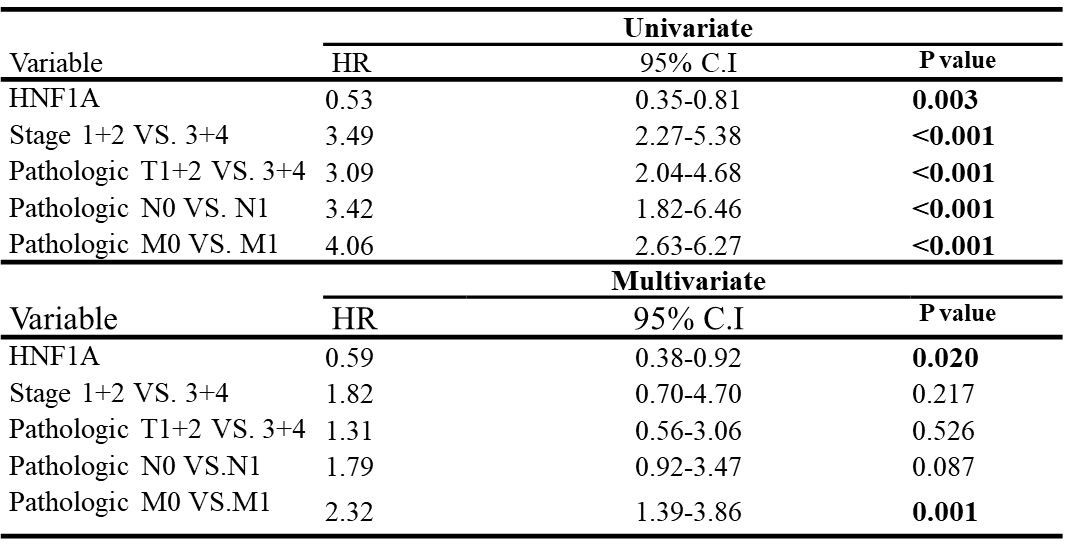


**Statistical significance *p*<0.05 shown in bold.**

**Supplementary Table 7.** Predicted binding sites for p53 identified on the promoter regions of MAOB, extending up to 2 kb upstream of the transcription start site

| Name | Relative score | Start | End | Strand | Sequence |
| --- | --- | --- | --- | --- | --- |
| p53 | 0.87681526 | 1528 | 1542 | - | ACATGCCCCAACATT |
| p53 | 0.81858677 | 1526 | 1543 | - | AACATGCCCCAACATTGT |
| p53 | 0.8177231 | 1526 | 1543 | + | ACAATGTTGGGGCATGTT |

**Supplementary Figure 1.** Different MAOB expression levels between 254 paired matched tumor and normal tissues, obtained from the Gene Expression Omnibus (GEO) dataset, GSE167093. *** *p*< 0.001.


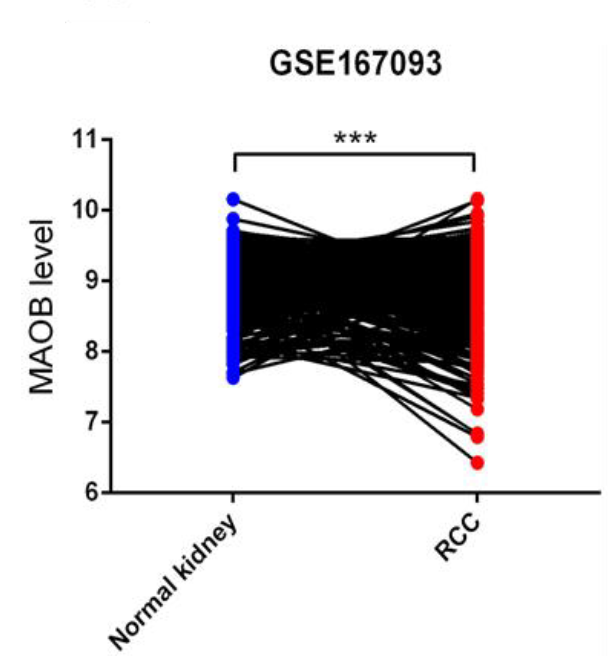


**Supplementary Figure 2.** MAOB levels in representative normal and RCC tumor tissues with various IHC scores.
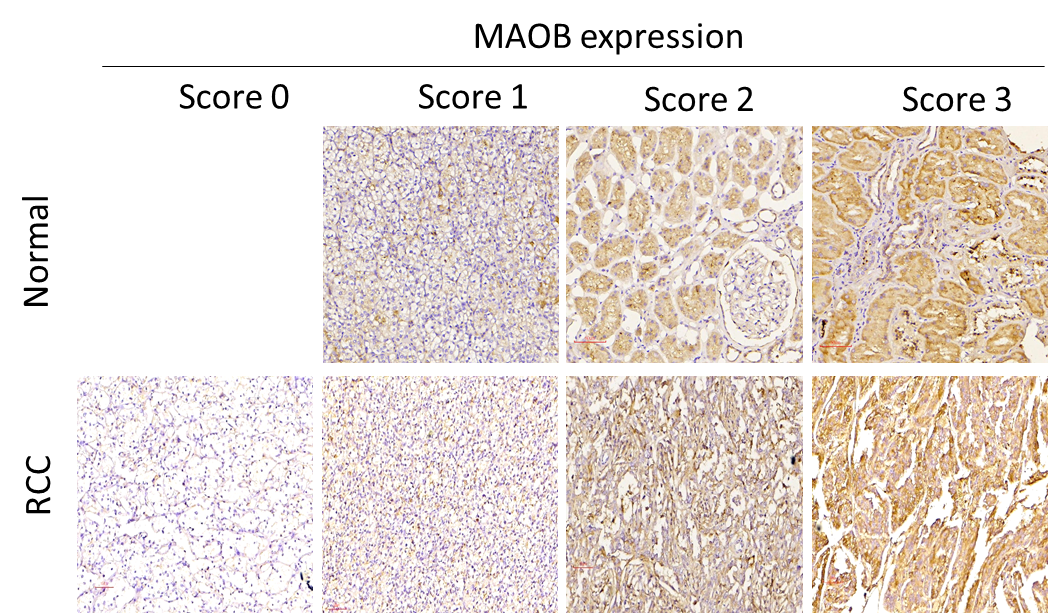


**Supplementary Figure 3.** Colony-forming ability of Caki-1/Neo and Caki-1/MAOB cells after 7 days of treatment with or without 10 μM of various MAOB inhibitors (selegiline, pargyline, or rasagiline). *** *p*<0.001 compared to the vector control group. ^###^ *p*<0.001 compared to the MAOB-overexpressing group.


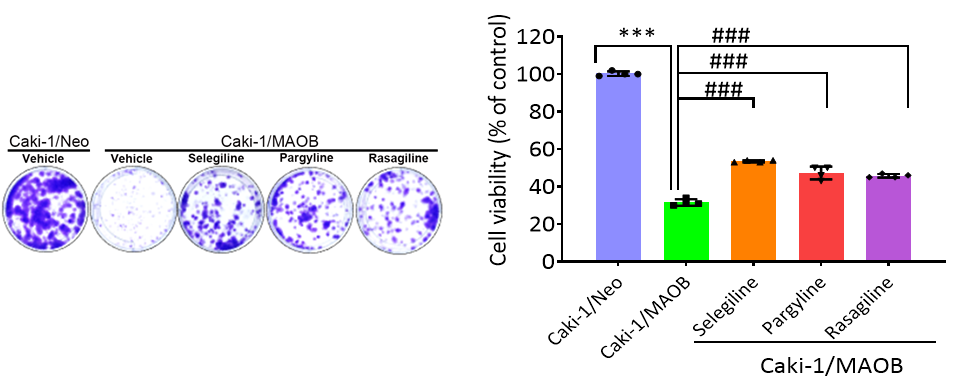


**Supplementary Figure 4.** Colony-forming ability of Caki-1 cells after transduction with wild-type MAOB, MAOB/Y435W, or a control vector. *** *p*<0.001 compared to the vector control group. ^###^ *p*<0.001 compared to the MAOB-overexpressing group.


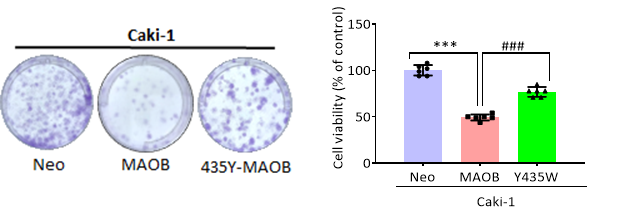


**Supplementary Figure 5.** Invasive and migratory abilities of Caki-1 and 786-O cells assessed following MAOB overexpression or knockdown. **p*<0.05, ** *p*<0.01, *** *p*<0.001 compared to the vector control group.


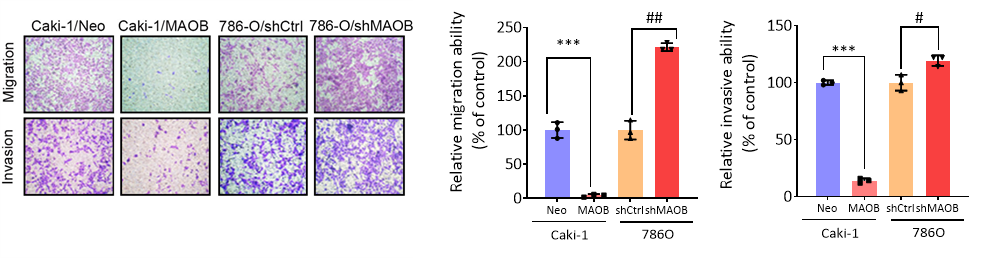


**Supplementary Figure 6.** Invasive and migratory abilities of Caki-1 cells following transduction with wild-type MAOB, the MAOB/Y435W mutant, or a control vector. *** *p*<0.001 compared to the vector control group. ^##^ *p*<0.01, ^###^ *p*<0.001 compared to the MAOB-overexpressing group.


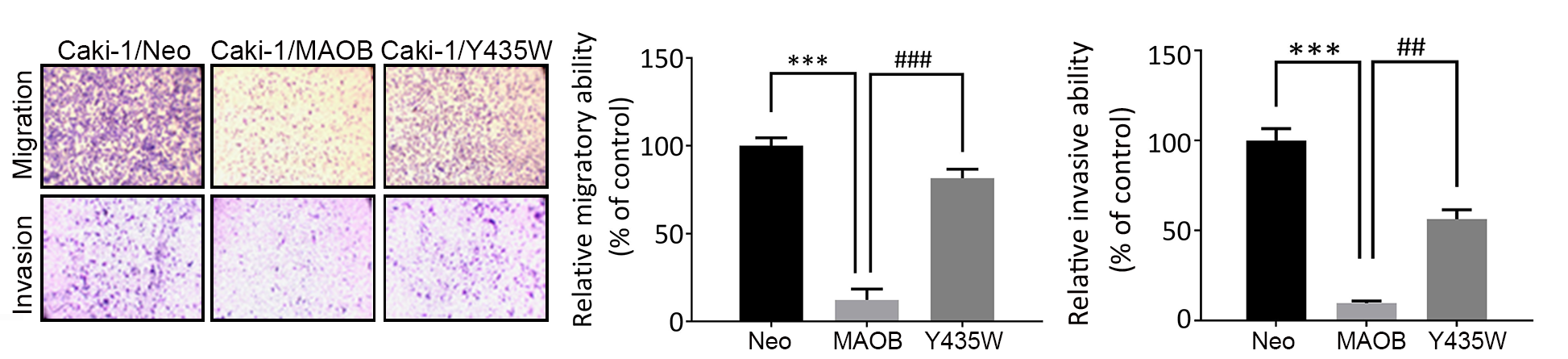

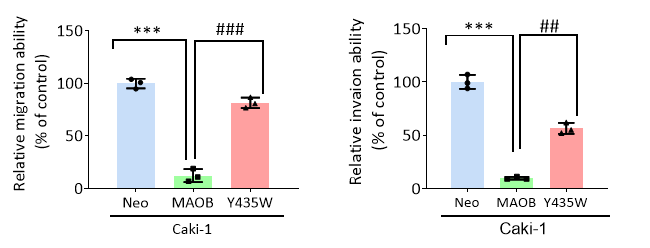


**Supplementary Figure 7.** Cytoplasm and nuclear H_2_O_2_ levels were measured by transfecting H₂O₂-sensitive biosensor HyPer7.2-NES and HyPer7.2-NLS respectively. The fluorescence ratio (F490/F420 nm) was calculated to quantify intracellular H₂O₂ levels in Caki-1/EV and Caki-1/MAOB cells treated with or without 10 μM selegiline or 5 mM of NAC. *** *p*<0.001 compared to the vector control group. ^###^ *p*<0.001 compared to the MAOB-overexpressing group.


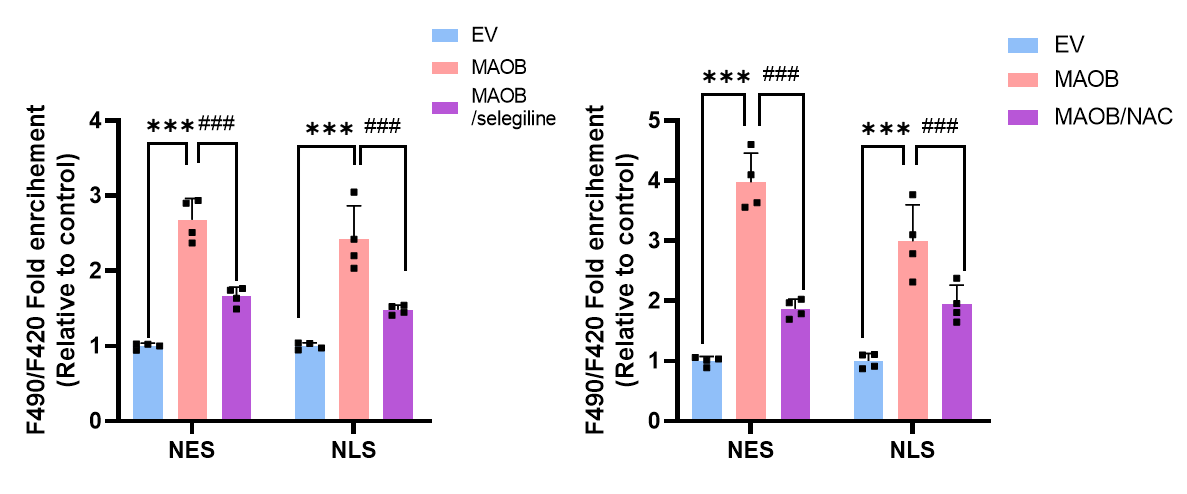


**Supplementary Figure 8.** Caki-1 cells overexpressing MAOB were treated with or without 5 mM GSH for 24 h, followed by assessment of lipid peroxidation using Liperfluo staining and flow cytometry. *** *p*<0.001 compared to the vector control group. ^###^ *p*<0.001 compared to the MAOB-overexpressing group.
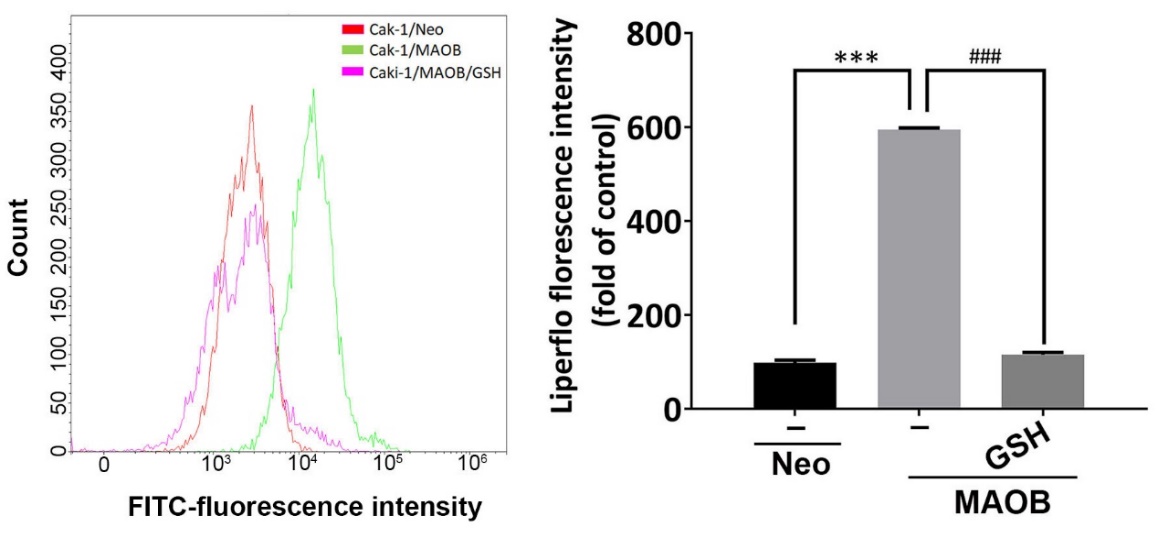


**Supplementary Figure 9.** Western blot analysis of MAOB, HO-1, and GPX4 expressions in Caki-1 cells overexpressing MAOB, with or without selegiline treatment.


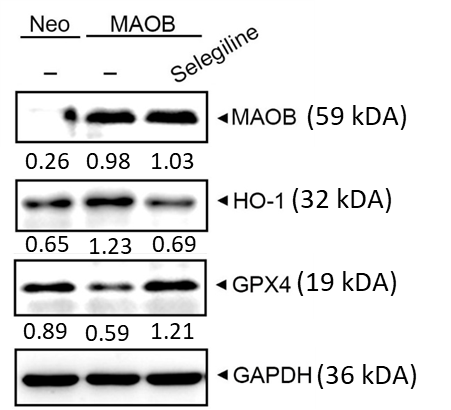


**Supplementary Figure 10.** Protein levels of MAOB, p53, and p21 analyzed by Western blotting in A498 cells following MAOB overexpression.


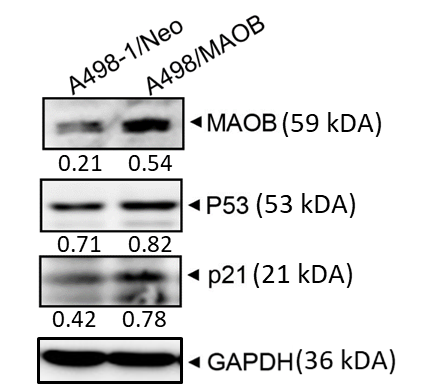


**Supplementary Figure 11.** Western blot analysis of MAOB, Puma, and Bak levels in MAOB-overexpressing Caki-1 cells treated with either PFT-α (10 μM) or vehicle for 24 h.


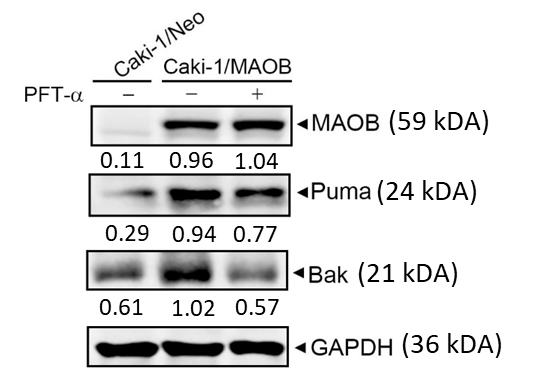


**Supplementary Figure 12.** Protein levels of PBRM1 analyzed by Western blotting in Caki-1 cells following MAOB overexpression.


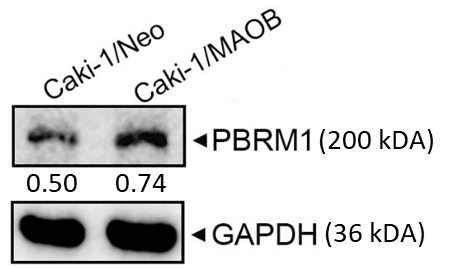


**Supplementary Figure 13.** Left panel: Dot plot illustrating a negative correlation between the methylation beta value of cg07390373 and *MAOB* gene expression in HNSCC. Right panel: Kaplan-Meier plot indicating that high methylation of cg07390373 is associated with poorer survival in patients with HNSCC.

**
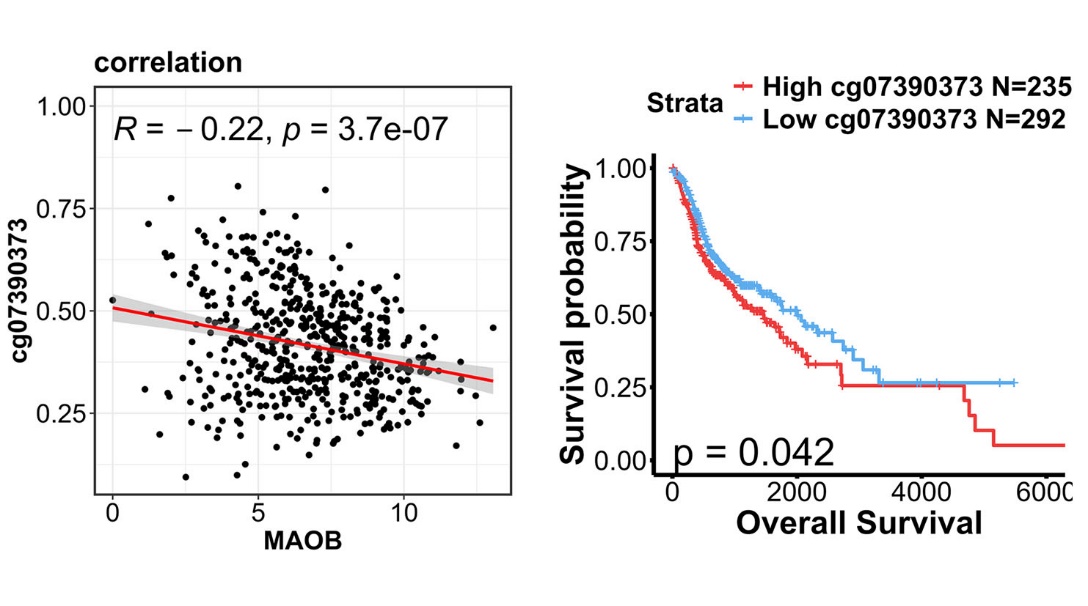
**

**Supplementary Figure 14.** SAS cells were treated with the indicated concentrations of 5-Aza for 24 h, and MAOB protein levels were assessed with a Western blot analysis.


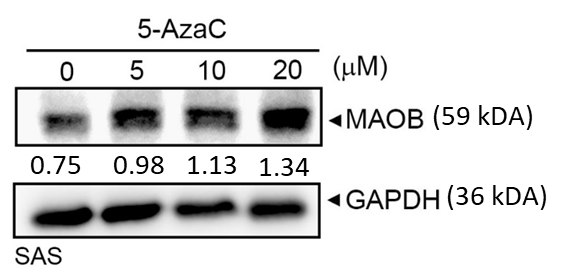

Supplement: Multimedia component 1 [file mmc1.docx]
