# Supplementary material for: MAOB promotes ROS-mediated DNA damage, triggering a cyclic MAOB-HNF1A-53BP1-p53 axis that suppresses the malignancy of clear cell renal cell carcinoma
Source: Redox Biol. 2025 Nov 25;88:103945. doi: 10.1016/j.redox.2025.103945 (PMC12719070; doi:10.1016/j.redox.2025.103945)

Fig2A

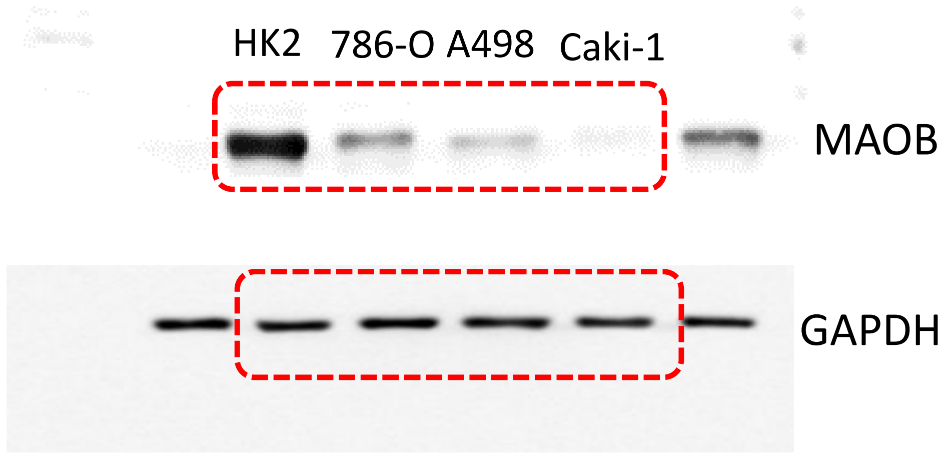
HK2 786-O A498 Caki-1
MAOB
GAPDH

Fig2B

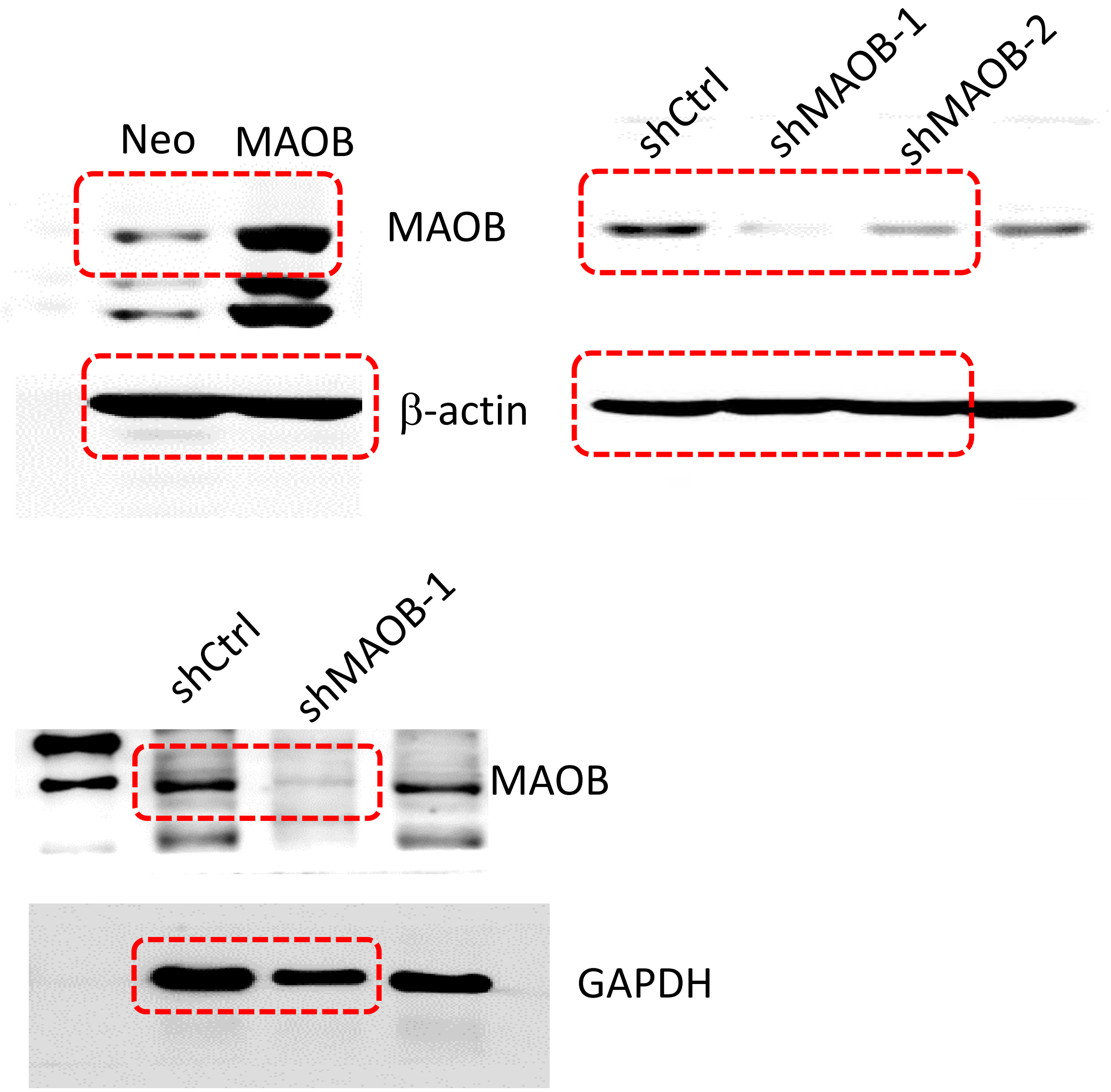
Neo MAOB
MAOB
β-actin
shCtrl shMAOB-1 shMAOB-2
shCtrl shMAOB-1
MAOB
GAPDH

Fig4B

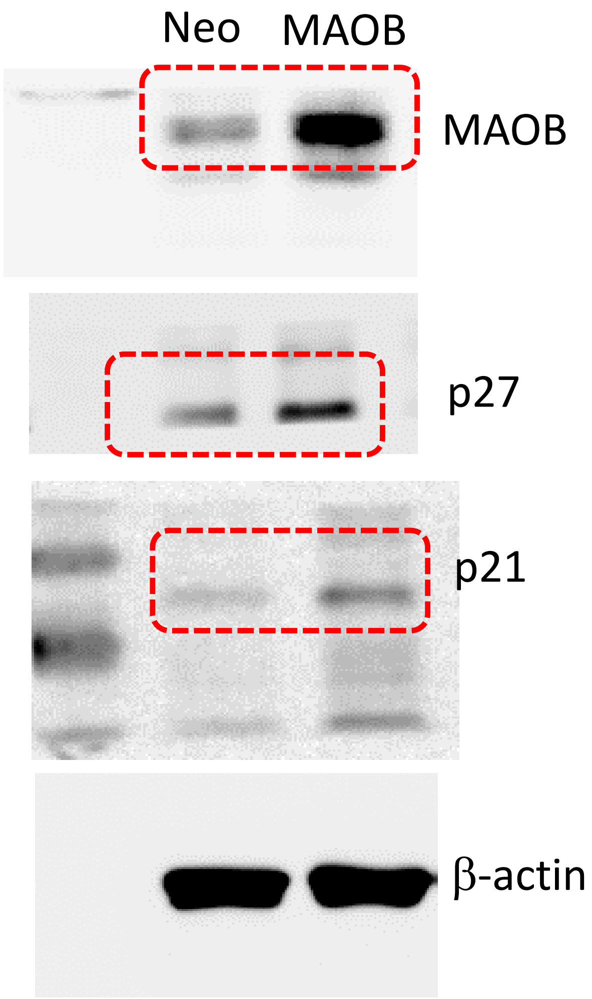

Fig4F

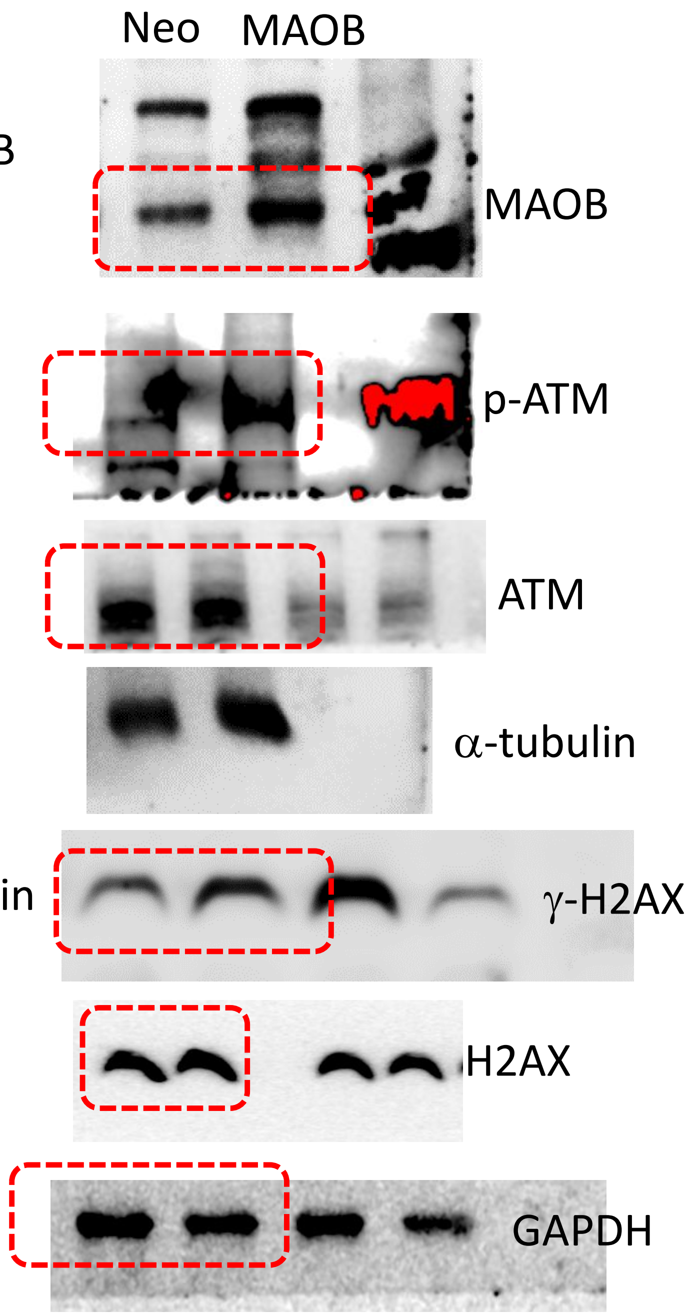

Fig4G

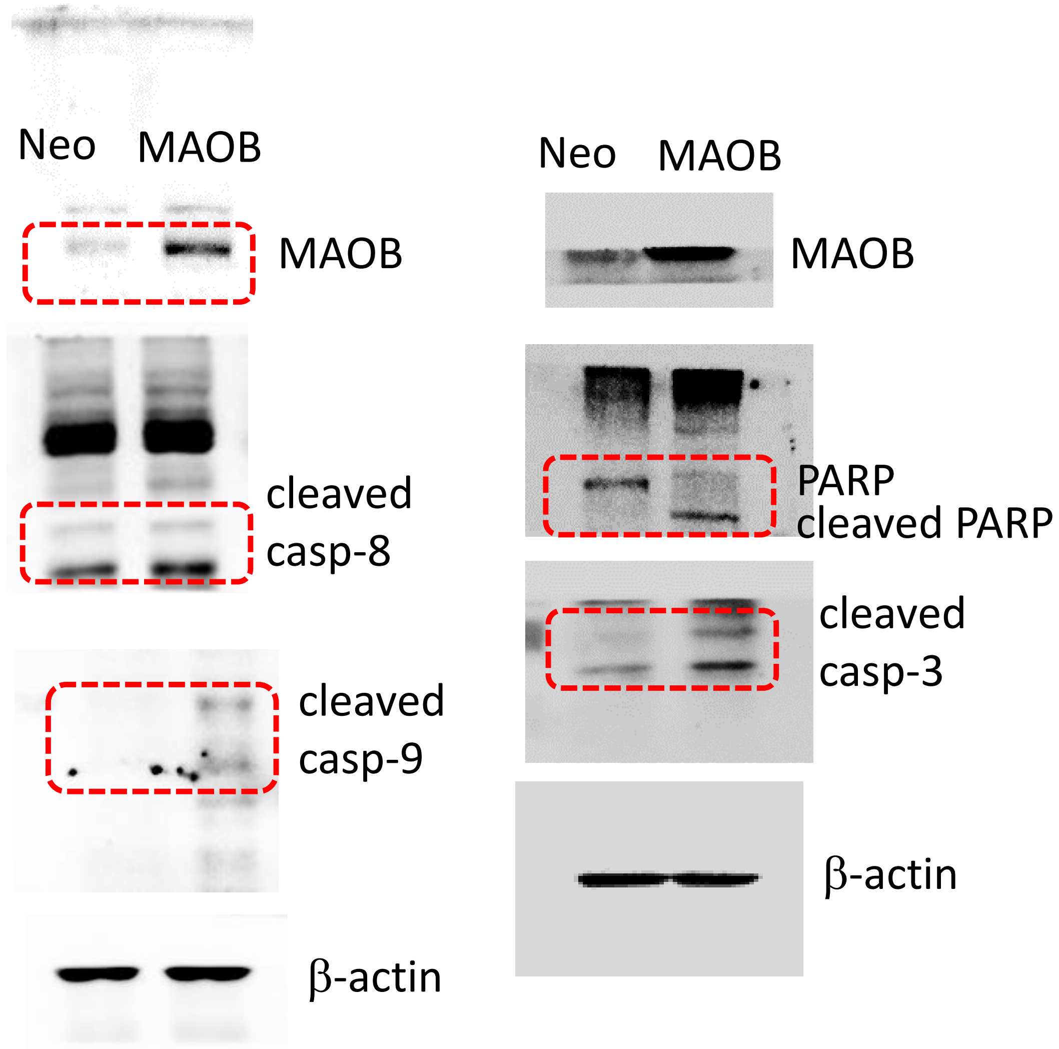

Fig4H

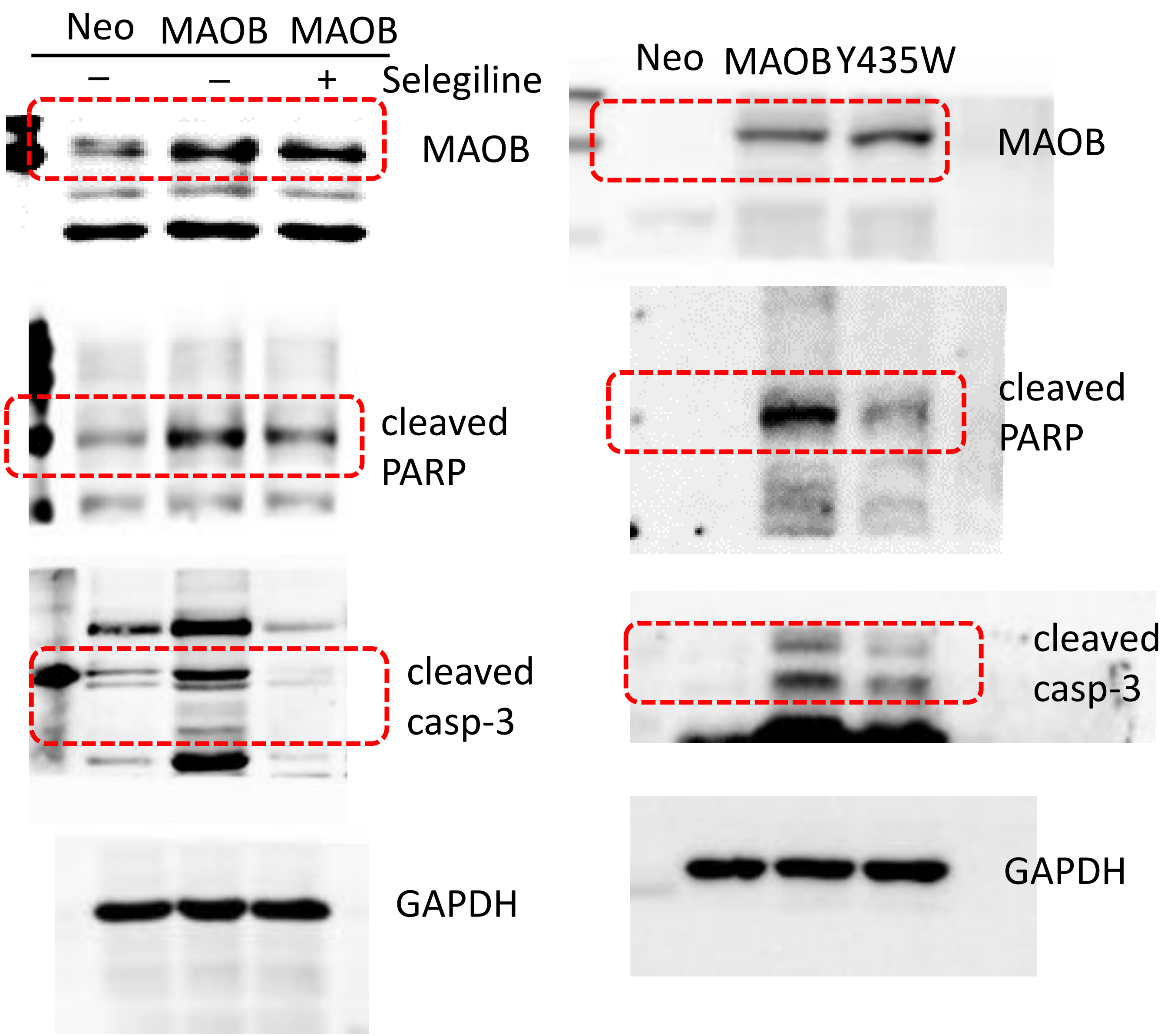

Fig4I

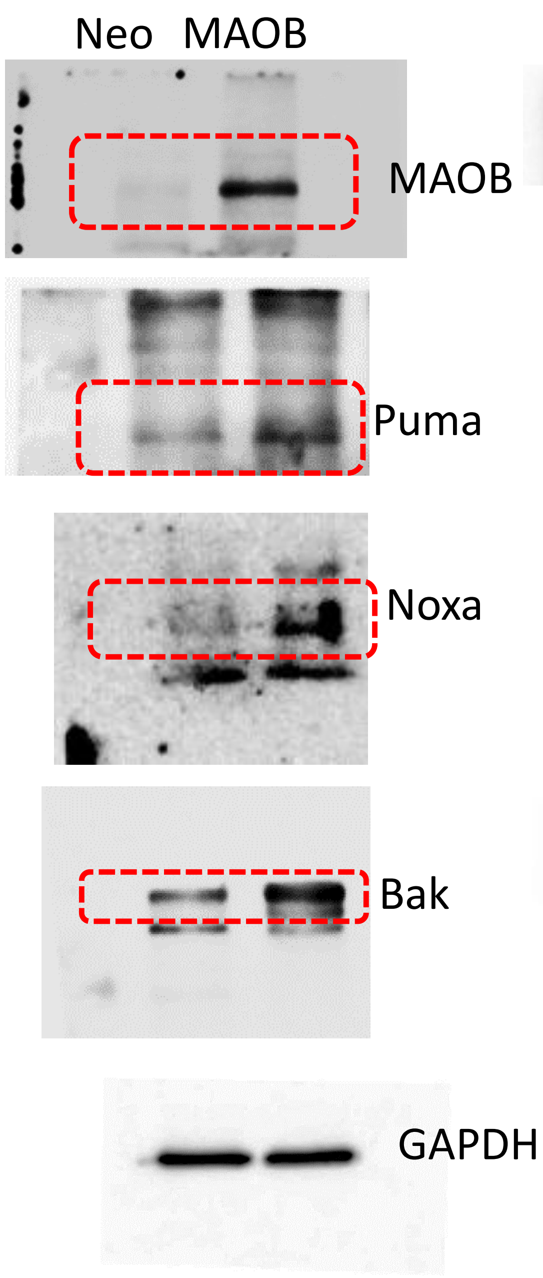

Fig4K

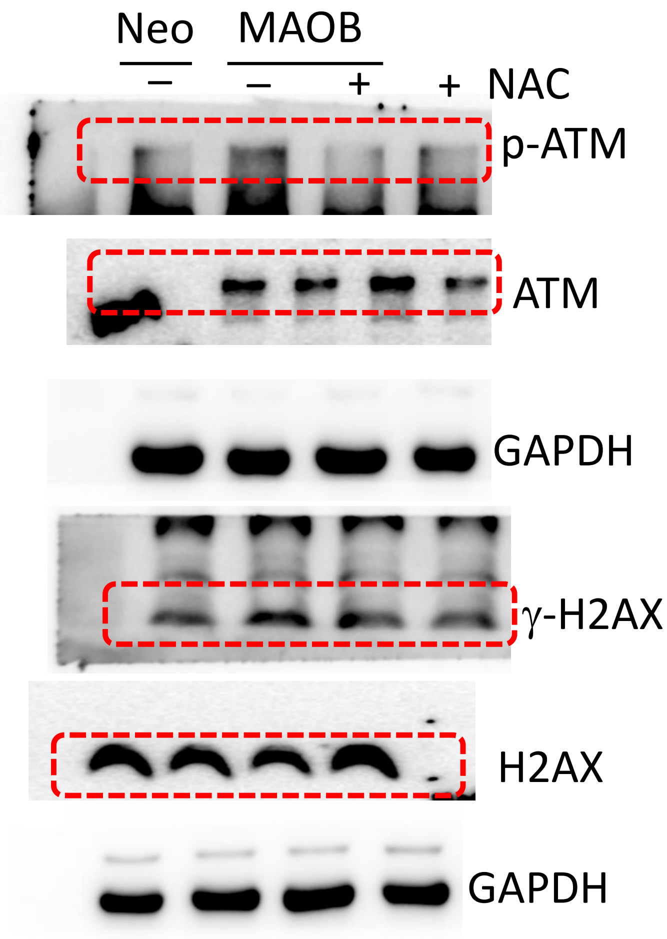

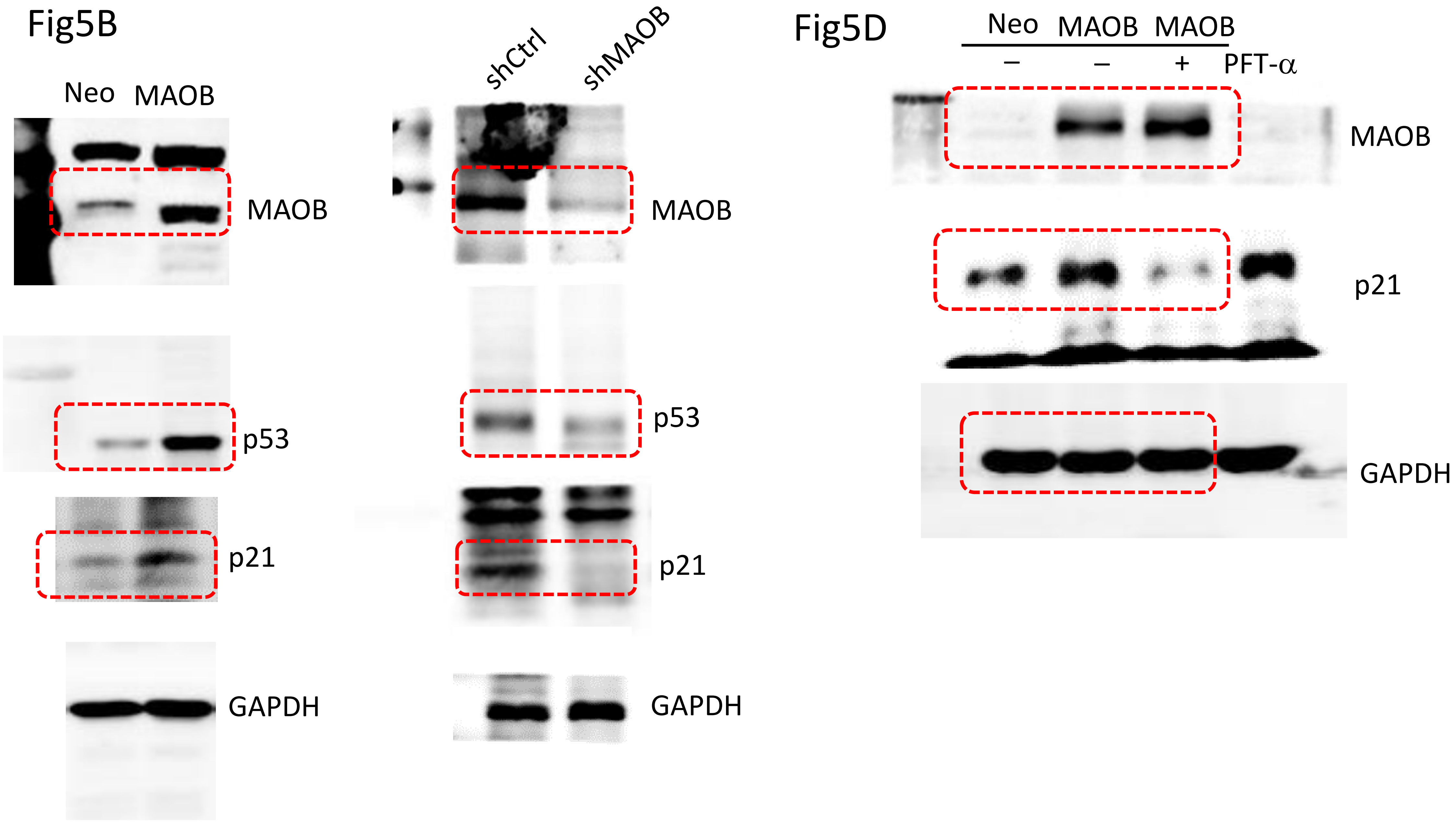
Fig5B
Neo
MAOB
MAOB
p53
p21
GAPDH
shCtrl
shMAOB
MAOB
p53
p21
GAPDH
Fig5D
Neo
MAOB
MAOB
–
–
+
PFT-α
MAOB
p21
GAPDH

Fig5F

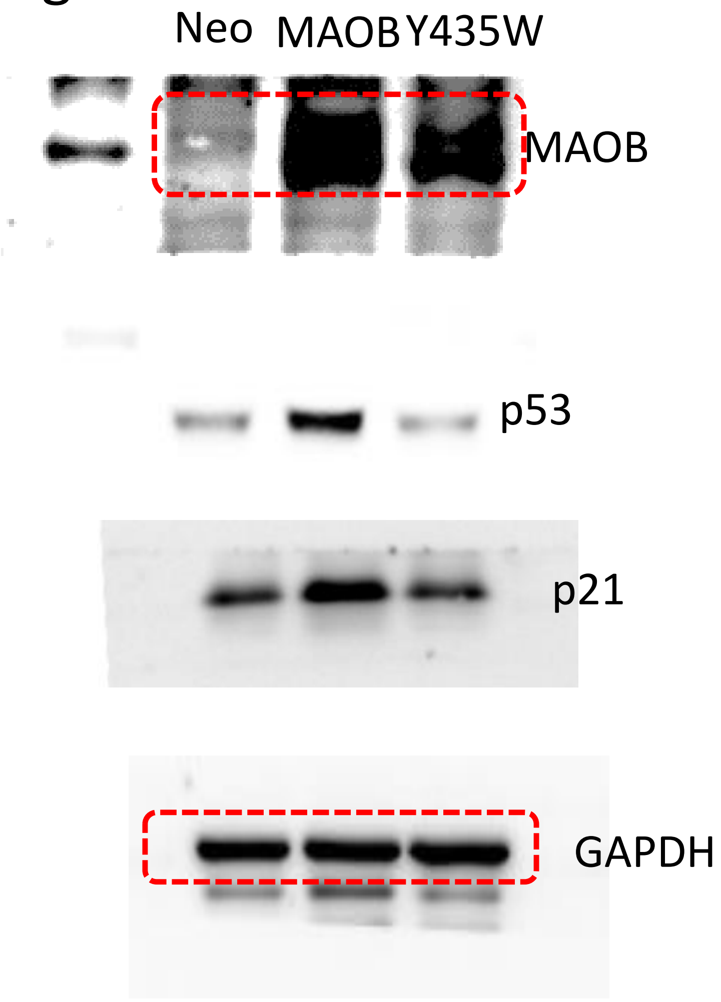

Neo
MAOB
Y435W
MAOB
p53
p21
GAPDH

Fig5H

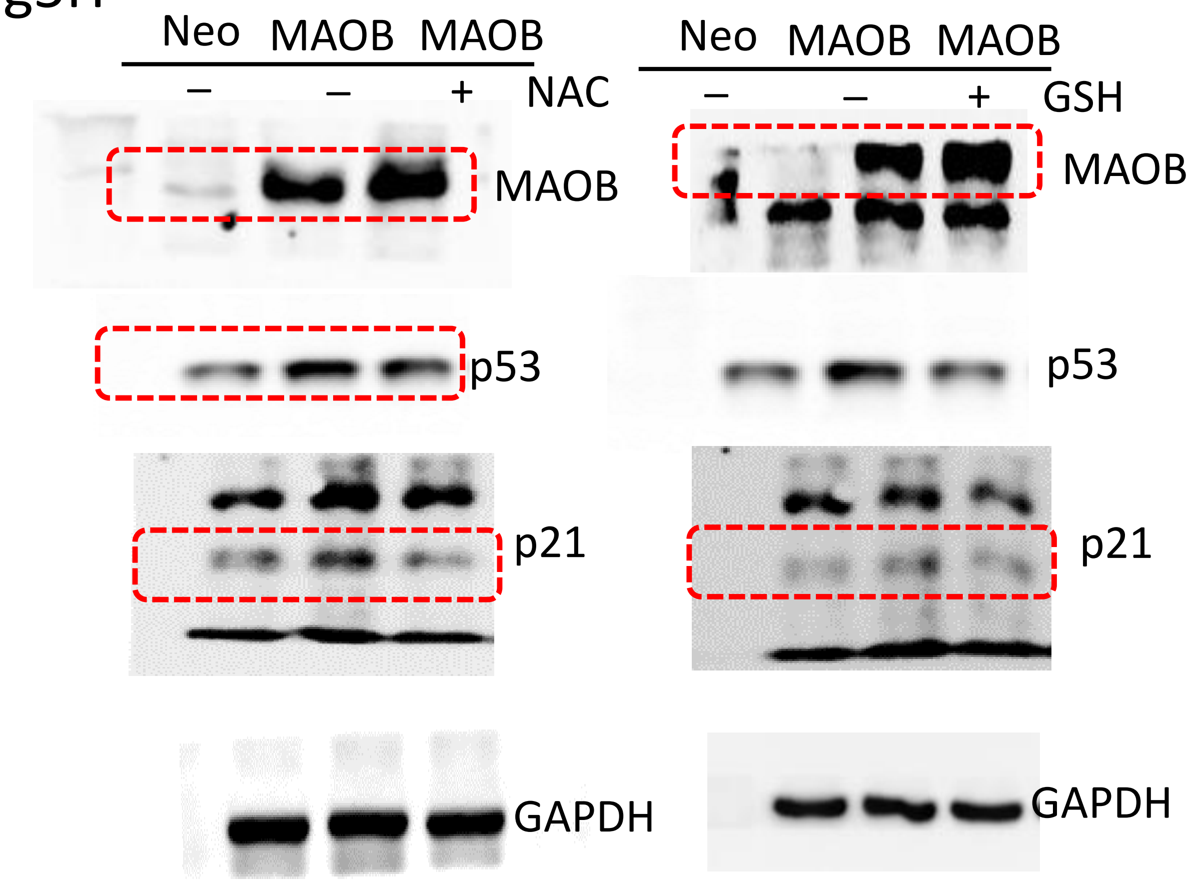

Neo
MAOB
MAOB
–
–
+
NAC
MAOB
p53
p21
GAPDH
Neo
MAOB
MAOB
–
–
+
GSH
MAOB
p53
p21
GAPDH

Fig5I

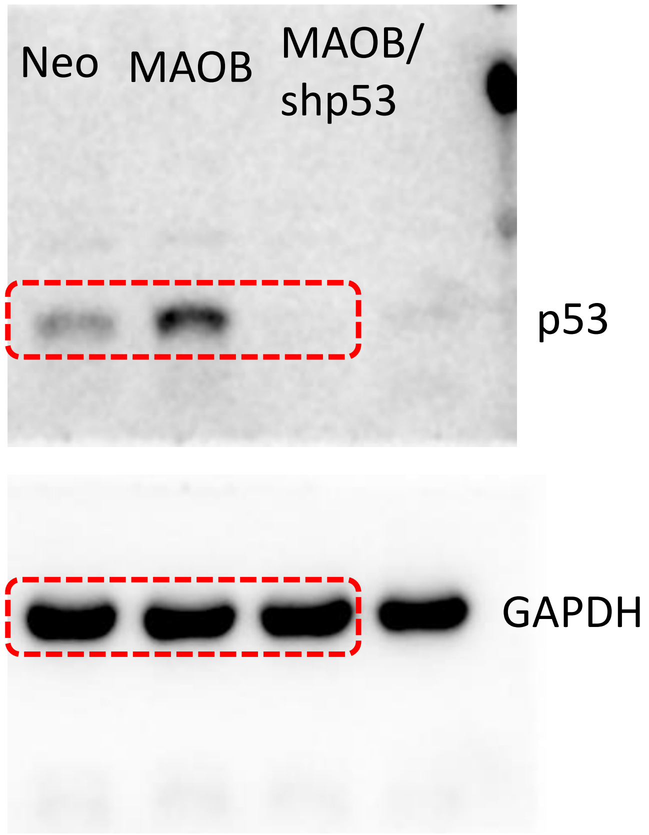
Neo
MAOB
MAOB/
shp53
p53
GAPDH

Fig5J

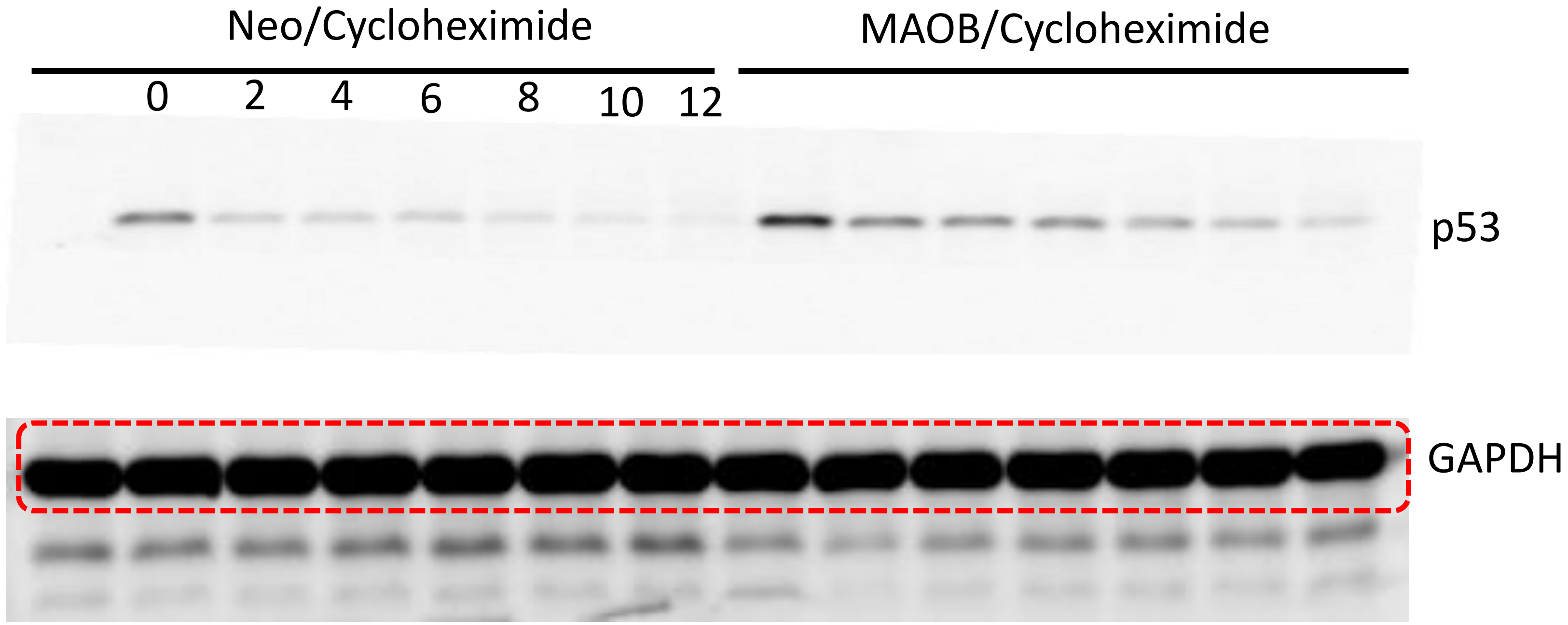
Neo/Cycloheximide
MAOB/Cycloheximide
0
2
4
6
8
10
12
p53
GAPDH

Fig5K

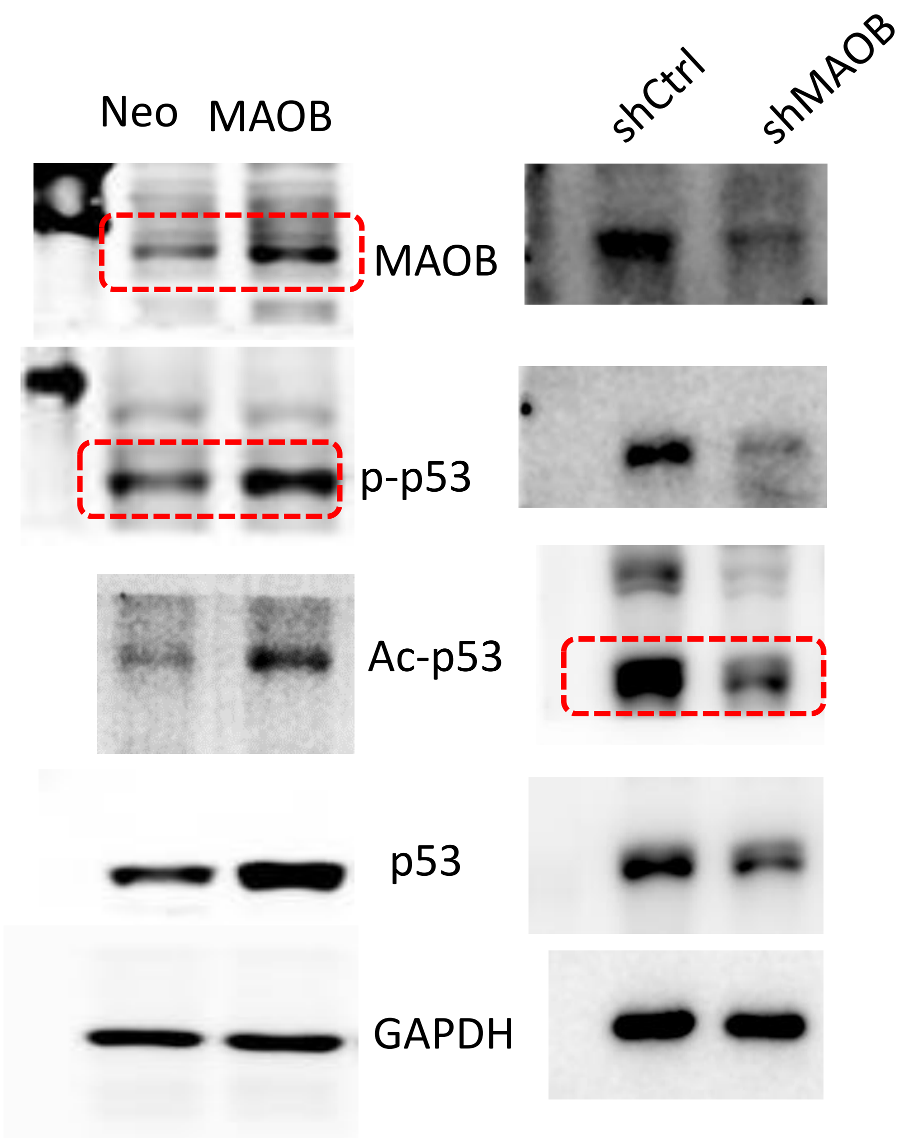

Fig6G

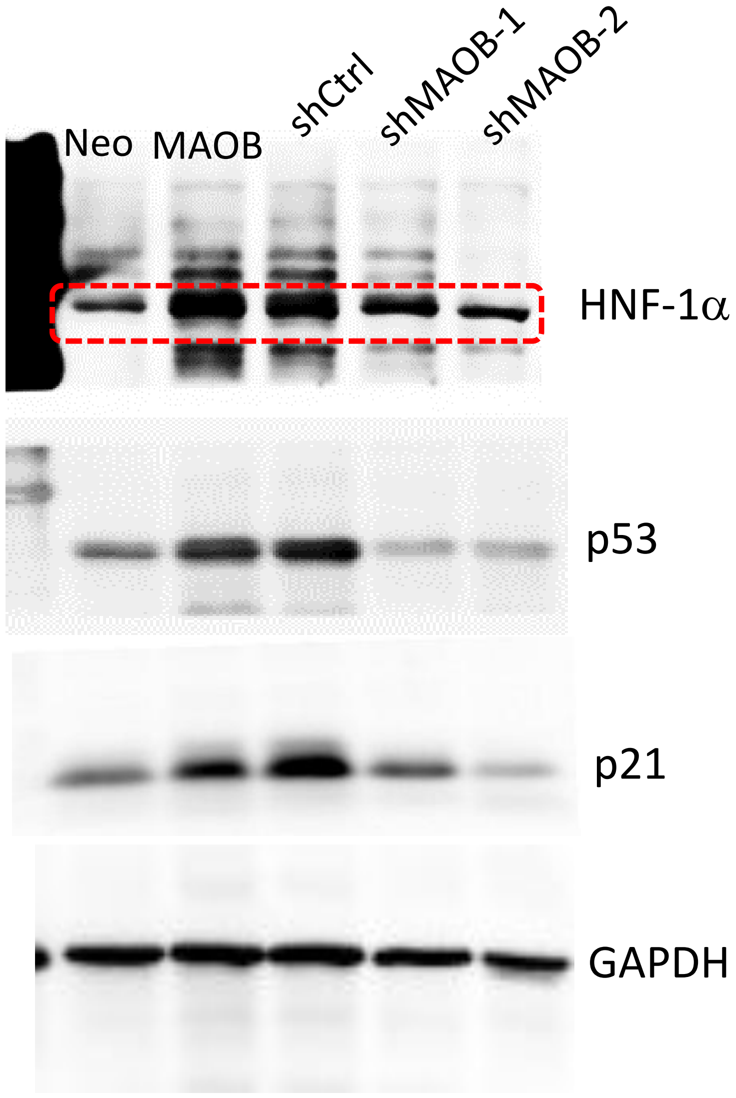

Fig6H

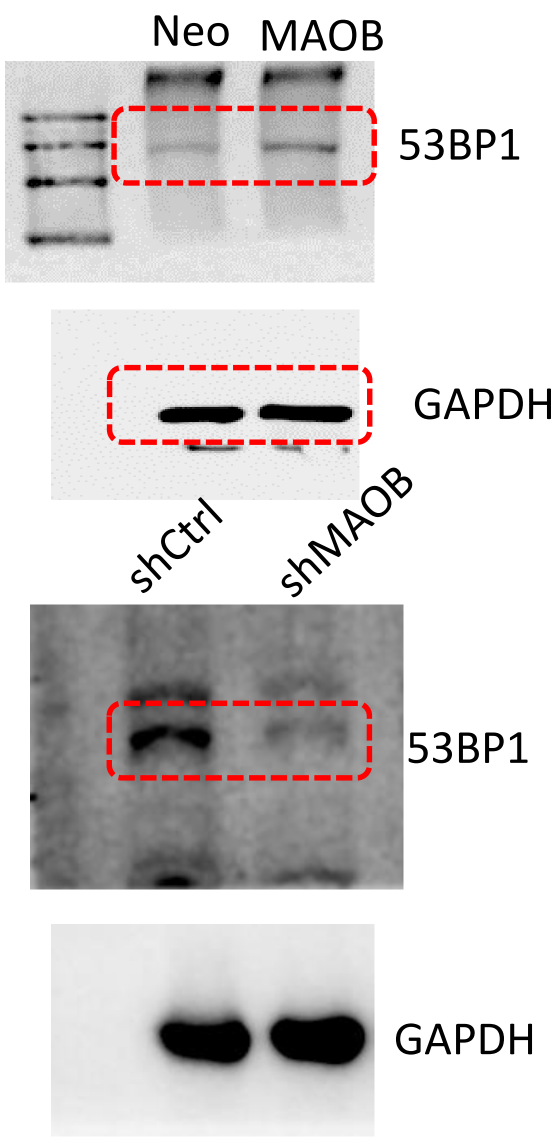

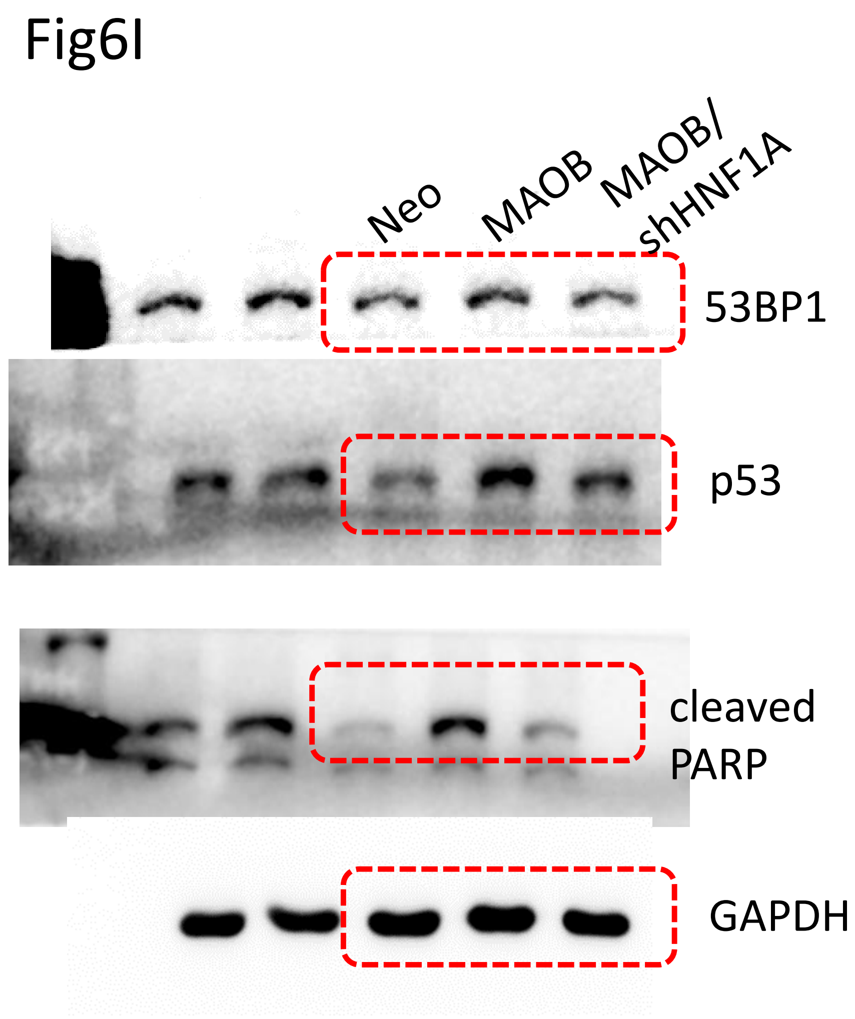
Fig6I
Neo
MAOB
MAOB/
shHNF1A
53BP1
p53
cleaved
PARP
GAPDH

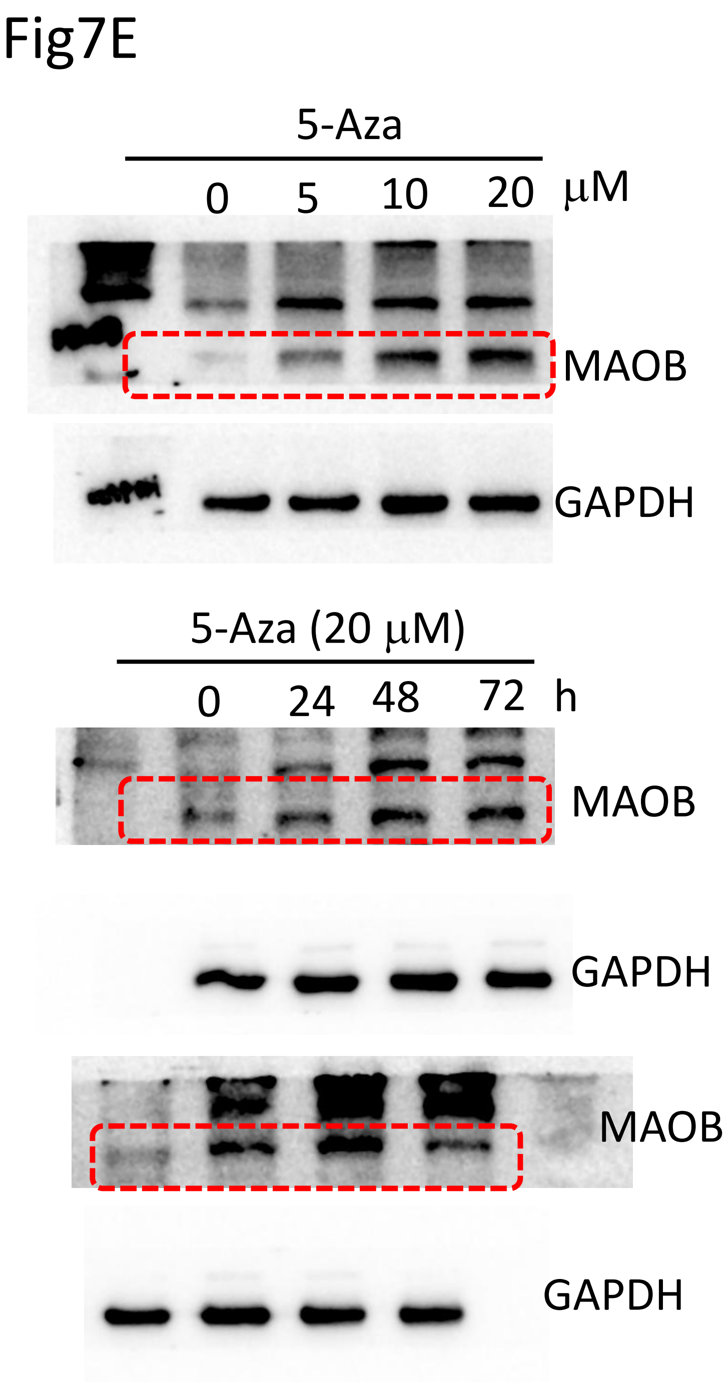
Fig7E
5-Aza
0
5
10
20
μM
MAOB
GAPDH
5-Aza (20 μM)
0
24
48
72
h
MAOB
GAPDH
MAOB
GAPDH

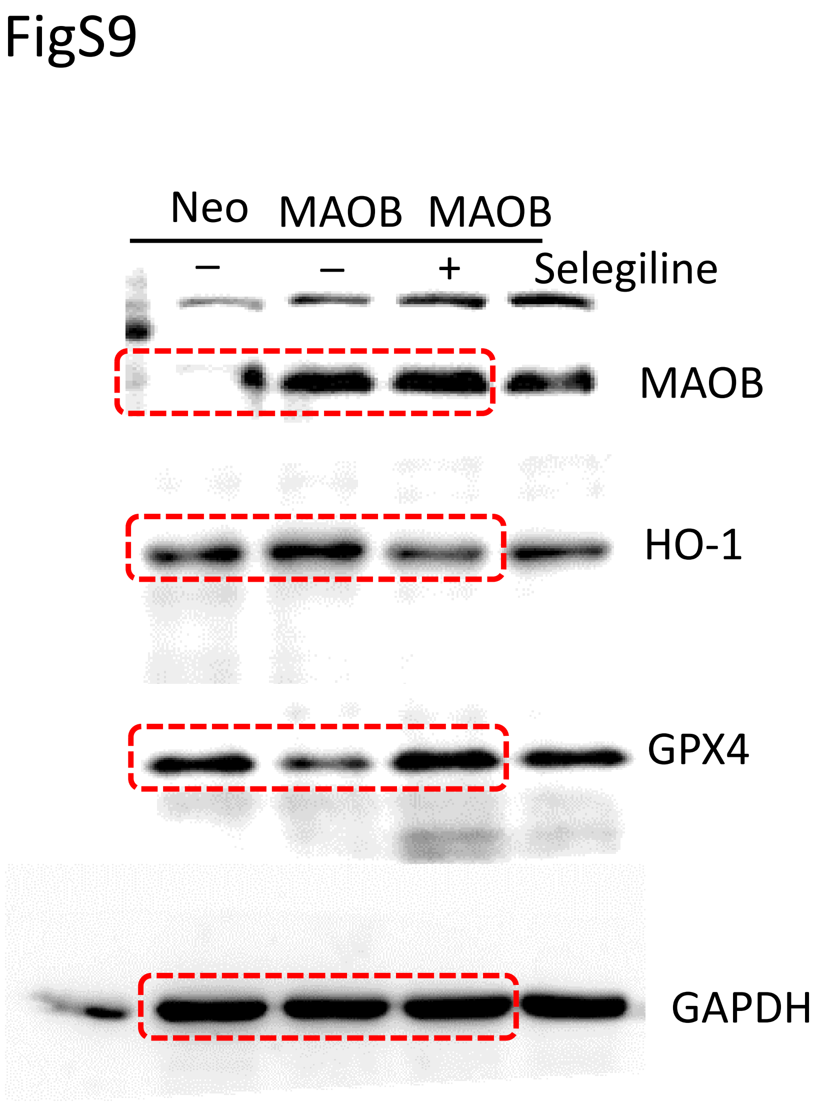
FigS9
Neo
MAOB
MAOB
–
–
+
Selegiline
MAOB
HO-1
GPX4
GAPDH

FigS10

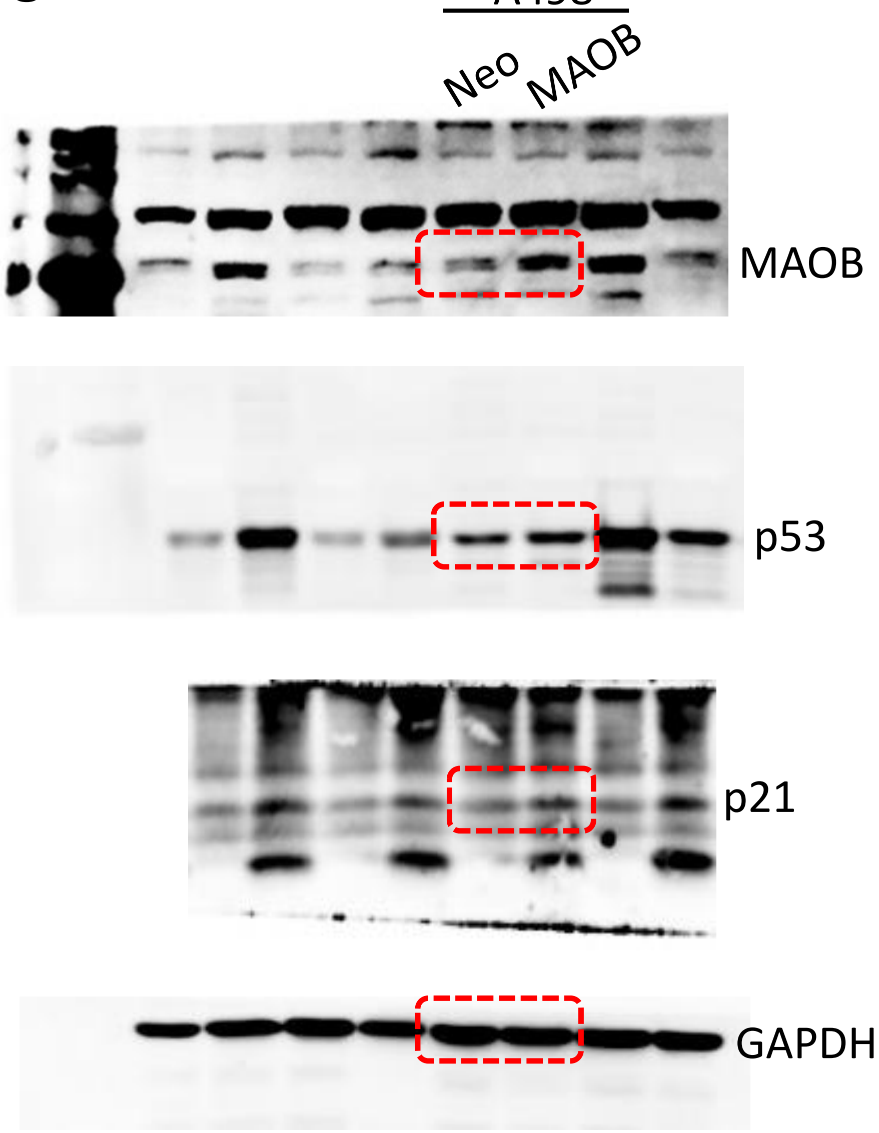

FigS11

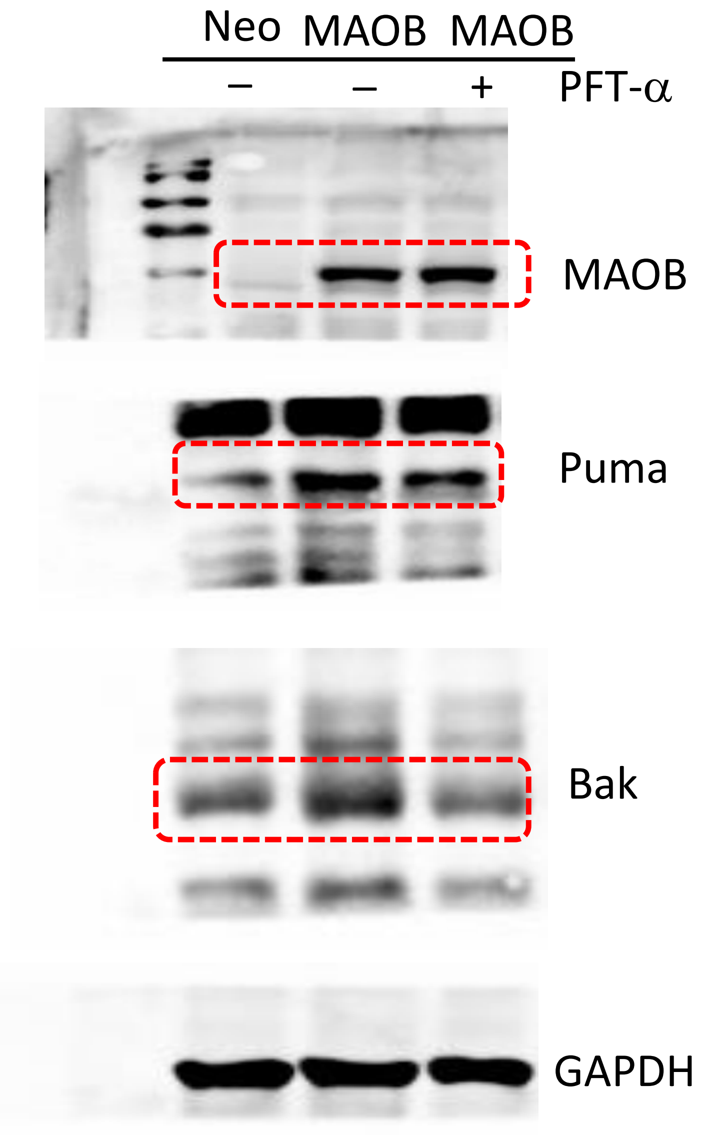

FigS12

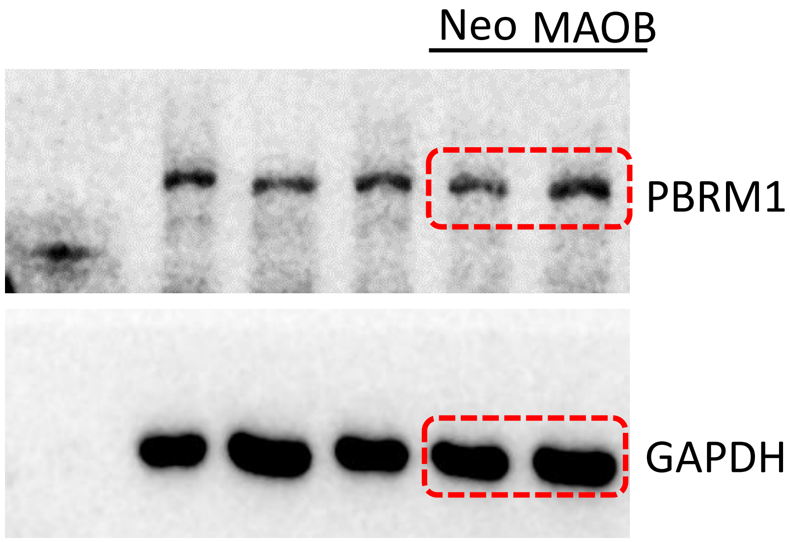

FigS14

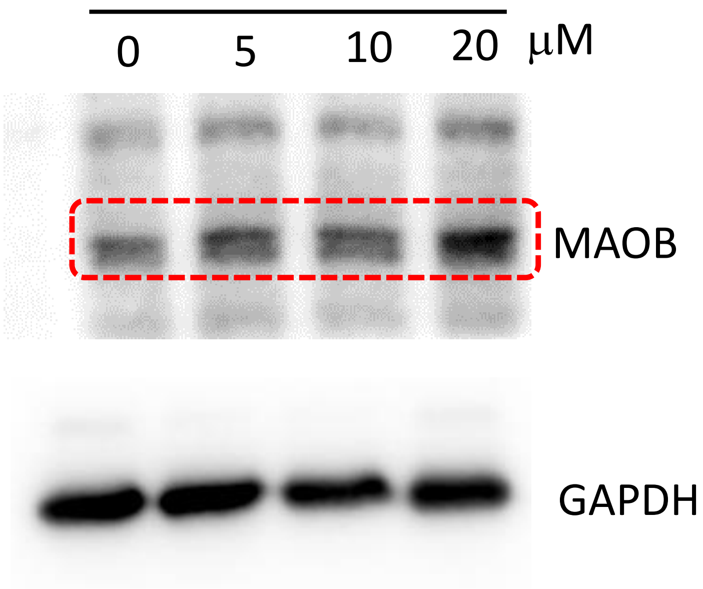

Supplement: Multimedia component 2 [file mmc2.pdf]
